# Autism spectrum disorder: pathogenesis, biomarker, and intervention therapy

**DOI:** 10.1002/mco2.497

**Published:** 2024-03-02

**Authors:** Hongbin Zhuang, Zhiyuan Liang, Guanwei Ma, Ayesha Qureshi, Xiaoqian Ran, Chengyun Feng, Xukun Liu, Xi Yan, Liming Shen

**Affiliations:** ^1^ College of Life Science and Oceanography Shenzhen University Shenzhen P. R. China; ^2^ Maternal and Child Health Hospital of Baoan Shenzhen P. R. China; ^3^ Shenzhen‐Hong Kong Institute of Brain Science‐Shenzhen Fundamental Research Institutions Shenzhen P. R. China

**Keywords:** autism spectrum disorder, biomarker, intervention therapy, molecular mechanisms, multi‐omics

## Abstract

Autism spectrum disorder (ASD) has become a common neurodevelopmental disorder. The heterogeneity of ASD poses great challenges for its research and clinical translation. On the basis of reviewing the heterogeneity of ASD, this review systematically summarized the current status and progress of pathogenesis, diagnostic markers, and interventions for ASD. We provided an overview of the ASD molecular mechanisms identified by multi‐omics studies and convergent mechanism in different genetic backgrounds. The comorbidities, mechanisms associated with important physiological and metabolic abnormalities (i.e., inflammation, immunity, oxidative stress, and mitochondrial dysfunction), and gut microbial disorder in ASD were reviewed. The non‐targeted omics and targeting studies of diagnostic markers for ASD were also reviewed. Moreover, we summarized the progress and methods of behavioral and educational interventions, intervention methods related to technological devices, and research on medical interventions and potential drug targets. This review highlighted the application of high‐throughput omics methods in ASD research and emphasized the importance of seeking homogeneity from heterogeneity and exploring the convergence of disease mechanisms, biomarkers, and intervention approaches, and proposes that taking into account individuality and commonality may be the key to achieve accurate diagnosis and treatment of ASD.

## Graphical abstract

Autism spectrum disorder result from a combination of genetic and environmental factors, with heterogeneity being a predominant characteristic. Despite this diversity, there exist convergent disease mechanisms and shared pathological features that provide a basis for diagnosis, treatment, and intervention, particularly in the context of stratification or subcategories. Besides, there remains optimism regarding the identification of specific mechanisms and the development of targeted diagnostic and therapeutic approaches.

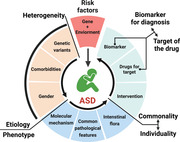

## 1 INTRODUCTION

Autism spectrum disorder (ASD) is a group of developmental neurological disorders characterized by early onset of abnormal social communication and restricted repetitive behaviors and interests. Since ASD was first discovered and defined, researchers have not stopped studying and exploring it (Figure S1).^1^, ^2^, ^3^, ^4^ Currently, the percentage of children with ASD has steadily increased since the 1970s, when it was less than 0.4%. It is currently estimated to be between 1% and 2%.^5^, ^6^, ^7^ The rate of ASD in 8‐year‐old children in the United States has increased from one in 44 in 2018 to one in 36 in 2020.^8^, ^9^ In China, the incidence of ASD in children aged 6−12 years is ∼0.7%.^10^, ^11^ As a result, ASD have attracted widespread societal attention.

**FIGURE 1:**
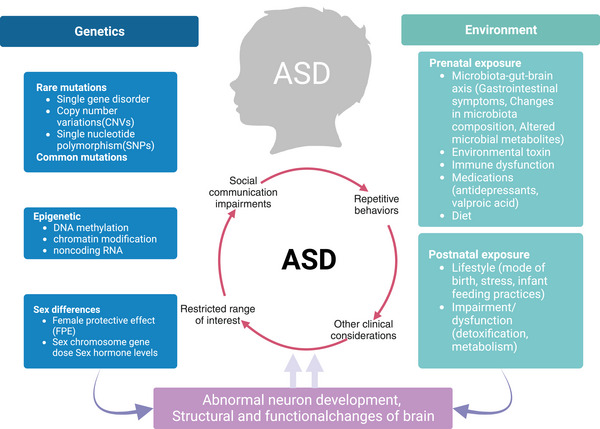
Potential influences of autism spectrum disorder (ASD). ASD is a heterogenous group of neurodevelopmental disorders characterized by social communication impairments, repetitive behaviors, restricted range of interest, and other clinical considerations. ASD is a multifactorial disease that involves the interactions of genetic and environmental factors. The genetic factors include genetics (single gene disorder, copy number variations and single‐nucleotide polymorphism), epigenetic (DNA methylation, chromatin modification and noncoding RNA), and sex differences factors (female protective effect and sex chromosome gene dose sex hormone levels). In contrast, the environmental factors comprise prenatal exposure (microbiota–gut–brain axis, environmental toxin, immune dysfunction, medications, and diet) and postnatal exposure (lifestyle and impairment/dysfunction). These factors lead to abnormal neuron development, changes in the structure and function of the brain, resulting in ASD.

The etiology of ASD is extremely complex. Twin studies suggest that genes play a key role in the pathogenesis of ASD, and its heritability estimates range from 64% to 91%.^12^ In families with children with ASD, the average rate of ASD recurrence is estimated to be 15%−25% for male newborns and 5%−15% for female newborns.^13^, ^14^ Besides, environmental factors are also implicated in the development of ASD, including prenatal/perinatal, microbial–gut–brain axis, and others. Prenatal/perinatal causes included maternal age >35 years, maternal characteristics of metabolic syndrome, use of antidepressant valproic acid (VPA) medications, and the effects of infection and inflammation.^15^, ^16^ Environmental factors can directly influence specific susceptibility genes, prompting epigenetic modifications such as DNA methylation and histone changes (phosphorylation and acetylation), which increase the risk of developing ASD.^17^ ASD arises from a complex interplay of genetic and environmental factors, leading to changes in brain structure and function that manifest as behavioral abnormalities (Figure 1).

Moreover, the heterogeneity of ASD impedes both pinpointing underlying mechanisms and tailoring effective therapies. Interestingly, the previous studies have shown that the function of ASD‐associated genes converges with the affected cell type^18^, ^19^, ^20^, ^21^, ^22^, ^23^ and that the affected brain has a characteristic molecular pathology.^22^ ASD‐specific molecular changes are mainly concentrated in central nervous system (CNS).^18^, ^19^, ^20^, ^21^, ^22^, ^23^ Besides, individuals with ASD have different comorbidities, but all share the same social communication deficits and repetitive stereotyped behavioral phenotypes, implying a common underlying biological mechanism among them.^24^ The heterogeneity of ASD does not preclude the possibility of finding common features or mechanisms that could lead to breakthroughs in the pathogenesis, diagnosis, and treatment of ASD. Efforts have been made to identify biomarkers, pathological mechanisms, and drug targets, and to explore the possibility of defining ASD subgroups by biological features.

In this review, we summarized the heterogeneity of ASD and explore its underlying disease mechanisms based on genes and multi‐omics studies. We focused on searching convergent disease pathways under genetic backgrounds and comorbidities. In addition, the mechanisms associated with common physiological and metabolic abnormalities and the gut microbiota were reviewed. An overview of research advances in ASD biomarkers was provided, and its role in early diagnosis was emphasized. Advances in behavioral interventions and pharmacological studies of ASD were also reviewed.

## 2 HETEROGENEITY OF ASD

Heterogeneity in etiology, phenotype, and outcome are hallmarks of ASD.^25^ These factors contribute to a clinical heterogeneity, which manifest as diverse deficits or impairments in behavioral features and communicative functioning. The remarkable heterogeneity of ASD complicates and diversifies the clinical diagnosis and the individualization of treatment for ASD, which involves a combination of multiple genes, environmental factors, and mental health disorders. Heterogeneity of genes, comorbidity in ASD, and gender bias contribute to the heterogeneity of ASD.^25^

### 2.1 The challenge from heterogeneity of genes

With the application of genome‐wide linkage and association analysis, copy number variant analysis, candidate gene resequencing and association analysis, and exome sequencing, many genes associated with ASD have been identified. Over 1200 genes have been recorded in the SFARI autism gene database (https://www.sfari.org/). More than 100 risk genes have been identified, including de novo mutations, genomic copy number variants, and single base mutations. Notably, children with ASD are genetic heterogeneous, with genetic variants detected in about 10%−20% of cases, but no single gene or mutation can cause more than 1% of cases,^26^ and genetic testing is still not available to accurately predict or diagnose ASD.

### 2.2 Comorbidity in ASD

In addition to core symptoms, children with ASD often have learning difficulties, intellectual disabilities (IDs), and other behavioral problems that may manifest as aggression, self‐injurious behavior, impulsivity, irritability, hyperactivity, anxiety, and mood symptoms.^27^ The severity of clinical symptoms and behavioral difficulties varies from person to person with autism and can have a severe or mild impact on daily life. Individuals with ASD are also more likely to have comorbid developmental and psychiatric problems such as attention deficit hyperactivity disorder (ADHD), anxiety and depression, ID, and specific disorders such as epilepsy, motor coordination, feeding difficulties, sleep disturbances, and gastrointestinal problems.^28^ About 29% of individuals with ASD are likely to have savant skills.^29^ The situation is complicated by changes in behavior and symptoms throughout development and maturity, as well as comorbidities that occur simultaneously.

### 2.3 Gender bias in ASD

Male preponderance is a highly replicated finding in ASD despite striking heterogeneity in symptoms and severity. The ratio of male to female prevalence was 4:1.^30^ In different studies, it has been reported that ASD is more prevalent in males possibly due to sex‐specific single‐nucleotide polymorphisms, single‐nucleotide variants, micro‐deletions, copy number variants, and proteins.^31^, ^32^, ^33^, ^34^, ^35^, ^36^ The findings of these studies have, however, not been consistently replicated in studies of the highly heterogeneous ASD.^37^ ASD preponderance and severity differences between males and females are explained by the female protective effect (FPE) theory.^26^ As part of the FPE, the greater variability model is included. Which asserts that males are more genetically variable, resulting in a higher incidence and decreased severity of ASD.^38^, ^39^ Additionally, the FPE incorporates a liability threshold model, which is based on the hypothesis that females who fulfill diagnostic thresholds for autism are more likely to carry mutations than males, and relatives of females with ASD tend to be more affected than relatives of males with autism.^40^ Other studies examining groups of people with ASDs and siblings of those with the disorder neither find an increase in the genetic burden of females with the disorder nor an increased incidence in female relatives of those with the disorder.^37^, ^41^, ^42^ It is possible that these differences can be attributed to the heterogeneity in the samples and the different methodologies employed. The future will require replication with larger groups.

## 3 POTENTIAL PATHOGENESIS OF ASD

Here, we reviewed the underlying mechanisms with the association of ASD risk genes, omics studies, ASD occurrence in different genetic backgrounds, and its common mechanisms between ASD and its comorbidities. We also summarized the mechanisms associated with important physiological and metabolic abnormalities, as well as gut microbiota.

### 3.1 Pathway networks associated with ASD risk genes based on SFARI database

Single gene mutations merely account for 1%–2% of autism cases and they act through distinct molecular pathways.^43^, ^44^ We gathered the ASD risk genes from SFARI database and categorized them into three groups based on risk level. The Gene Ontology (GO) analysis was conducted on three groups, respectively. In the first set, most of risk genes were enriched in histone modification, cognition, as well as regulation of transporter activity pathway. Regulation of neurological system process, synapse organization, and social behavior pathways were placed in a prominent position within pathway network (Figure S2A). These results implicated that impairment of cognition is the most obvious character. Individuals with autism spectrum conditions or rare mutation related to ASD have profound impairments in the interpersonal social domain.^45^, ^46^, ^47^, ^48^ In the second set, a majority of the risk genes exhibited enrichment in modulation of synaptic transmission, synapse organization, and learning or memory (Figure S2B). Additionally, some pathways involved human traits and actions were found, including learning or memory, social behavior, mating, circadian rhythm, sleep, and locomotory behavior. The change of these human action may be potential indication for ASD.^49^, ^50^, ^51^, ^52^, ^53^ In the third set, many risk genes were enriched in cellular response to peptide, regulation of cell growth, and modulation of synaptic transmission (Figure S2C).

### 3.2 Multiple omics revealed pathological mechanism of ASD

Omics techniques allowed an in‐depth study of ASD from a wide range of samples. The advantage of omics approaches is that they provide a complete overview of biological “features” (genes/transcripts/proteins/metabolites). It provided the most appropriate stratification of diseases or identification of new biomarkers. Meanwhile, multi‐omics can integrate information across different populations, validate them against each other, identify key genes, proteins and metabolic pathways, explore pathological mechanisms, and provide a scientific basis for the disease diagnosis and treatment. Here, we reviewed the omics studies related to ASD and the signaling pathways, in particular the convergent signaling pathways (Table S1) which associated with synaptic dysfunction, glutamatergic and GABAergic synapse imbalance, and postsynaptic density (PSD), as well as important physiological and metabolic abnormalities.

#### 3.2.1 The signaling pathways of synaptic dysfunction

The main signaling pathways involved in synaptic dysfunction include phosphatidylinositol 3‐kinase/Protein kinase B/Mammmalian target of rapamycin (PI3K/Akt/mTOR) signal and abnormal autophagy, extracellular signal‐regulated kinase/mitogen‐activated protein kinase (ERK/MAPK) signal, Janus kinase and microtubule interacting protein 1 (JAKMIP1) pathway, and calcium signaling. Among them, dysregulation of the PI3K/Akt/mTOR pathway was considered as a point of convergence ASD.^54^, ^55^, ^56^ mTORC1 severed as a key role to tightly coordinates synaptic signaling pathways downstream of glutamate and neurotrophic receptors.^57^ An unbiased proteomic showed that a brief repression of mTORC1 activity causes a significant remodeling of proteins resided in the PSD.^58^ A rat fetal brain transcriptome demonstrated prominent maternal immune activation (MIA)‐induced transcriptional dysregulation of mTOR and EIF4E‐dependent signaling.^59^ The significant proteins from S‐nitrosylation proteomics could be enriched in mTORC1 upstream pathway in InsG3680(+/+) ASD mouse models.^60^ DEPs from frontal cortex (FC) and hippocampus of Tsc1+/− mouse model were involved in myelination, dendrite, and oxidative stress, an up‐regulation of ribosomal proteins and the mTOR kinase.^61^ In addition, a leukocyte transcriptomics identified a perturbed gene network involved with PI3K/AKT and its downstream pathways such as mTOR, autophagy, viral translation, and FC receptor signaling were enriched from 1−4‐year‐old male toddlers with ASD or typical development.^62^ Likewise, autophagy dysfunction meditated by PI3K/AKT/mTOR pathway is a causative factor for ASD.^55^, ^63^, ^64^

Accumulating evidence suggested ERK/MAPK signaling as a downstream mediator of divergent genetic mutations linked to certain forms of autism.^65^, ^66^, ^67^, ^68^ It also could be a converge on mTOR signaling pathway.^69^ A global down‐regulation of the MAPK/ERK pathway and decrease in phosphorylation level of ERK1/2 were found in Fmr1‐KO cell lines.^70^, ^71^ NMDA NR1‐knockdown mouse show the abnormalities of ERK signaling pathway in FC and hippocampus.^72^ MAPKAPK3 and MRPL33 in human blood were associated with a higher risk of ASD, and MAPK/ERK signaling pathways and mitochondrial dysfunction play key roles in the pathogenesis of ASD.^73^

The alteration of JAKMIP1 could be found in individuals with distinct syndromic forms of ASD, fragile X syndrome, and 15q duplication syndrome.^74^ A previous study found that CYFIP1 play a role in regulating two dysregulated genes, JAKMIP1 and GPR155 compared the mRNA expression profile in lymphoblastoid cells from autism.^75^ An enriched network from interactome showed that JAKMIP1 interacted with proteins related to signaling and interaction, nervous system development and function, and protein synthesis. Notably, its loss affected neuronal translation and glutamatergic N‐methyl‐D‐aspartate receptor (NMDAR) signaling.^74^

Calcium signaling has a prominent effect on pathogenesis of ASD.^76^ An action of calcium ion plays an essential role for neurodevelopment.^77^ ERK signaling has also been found to be greatly linked to calcium channels to cause abnormal synaptic functions, chromatin remodeling, and ion channel activity.^78^, ^79^ Ca^2+^/calmodulin‐dependent protein kinase II is considered as key node in synaptic plasticity of ASD.^80^ Its interactome identified proteins related to NMDARs, synaptic scaffolds, myosins, tubulin and microtubules, actin cytoskeleton, ribosome and translation, mitochondria, and others.^81^ Synaptic fraction contained more CaMKII‐associated proteins including scaffolding, microtubule organization, actin organization, ribosomal function, vesicle trafficking, and others.^81^ Activated CaMKII phosphorylates multiple substrates in the PSD, including scaffold protein PSD‐95, α‐amino‐3‐hydroxy‐5‐methyl‐4‐isoxazolepropionic acid receptor (AMPA) receptor targeting subunit stargazing, and proteins involved in cytoskeleton rearrangement.^82^

#### 3.2.2 Imbalance between glutamatergic and GABAergic synapse

Accumulating evidence supported a hypothesis that the imbalance between excitation and inhibition (E/I) caused by changes in the availability of glutamate and/or GABA signal transmission contribute to pathological synaptic transmission and neural circuits in ASD.^83^, ^84^, ^85^, ^86^, ^87^ A broad transcriptomics from postmortem samples with ASD demonstrated that both rare and common ASD‐associated genetic variation converge within a down‐regulated synaptic signaling.^88^ Previous study found a decrease of AMPA‐type glutamate receptors, glutamate transporters, and density of GABAA receptors in the cerebellum and anterior cingulate cortex of ASD.^89^ An orthogonal selected reaction monitoring assays validated the proteomics results in NMDA NR1‐knockdown mouse to show the abnormalities of synaptic long‐term potentiation and myelination in FC and hippocampus.^72^ Another proteomics study showed up‐regulation of glutamatergic ion channels and down‐regulation of neurofilament proteins in ASD brain.^90^ Similarly, a cortical transcriptome of ASD exhibited analogous cortical–striatal hyperconnectivity at the protein level with mTOR or TSC2.^91^ A single‐cell transcriptomics from *Chd8* heterozygote mice strengthen the E/I balance hypothesis of ASD in general.^92^

Interestingly, previous metabolomics studies found that ASD often suffer from dysregulated amino acid metabolism and glutamate urinary level was lower compared with their unaffected siblings.^93^, ^94^ The reduced pyridoxal phosphate in urine from ASD children implicated the dysregulation of biotransformation of glutamate into GABA.^95^ Similarly, a strongly reduced glycine level would primarily affect NMDAR excitatory tone, overall impairing downstream glutamatergic, and GABAergic transmissions.^96^

#### 3.2.3 Essential role of postsynaptic density in neural communication

The PSD of synapses is a wide range of scaffolding proteins, receptors, and signaling molecules that acts as a switchboard of neurotransmitter molecular and have strong association to ASD.^97^, ^98^ Glutamate receptor levels could be regulated by endocytosis of PSD scaffolding proteins.^99^ In general, E/I balance required the integrity of PSD to transmit signal between neuros.^100^, ^101^, ^102^ Several genes encoding PSD have been identified disruptive mutations in psychiatric disorder patients, including ASD.^98^, ^103^

Synaptic protein/pathways resource (SyPPRes) was identified as the prioritization of ASD risk factors across 41 in vivo interactome, which show a larger number of shared protein associations to Psd95/Dlgap1/Shank3 indicating a role of core–PSD scaffolds interactions.^104^ The alteration of macromolecular complex proteins such as SHANK3 can cause ASD.^105^ To quantify the proteins in PSD fractions, the most altered levels of proteins exhibiting ionotropic glutamate receptor activity, cell–cell signaling, and cytoskeleton organization as the results of SHANK3 deficiency.^106^ A zebrafish embryo model of ASD induced by VPA showed the significant decrease of *Shank3* in transcriptome.^107^ Striatal regions of *Shank2*‐mutant mice showed distinct patterns from transcriptomic including synapse, ribosome, mitochondria, spliceosome, and extracellular matrix.^108^ The transcriptomic from hippocampal showed strongly enriched GO terms associated with PSD, synapse, and postsynaptic membrane.^108^ Other omics studies related to ASD risk genes have achieved similar results, such as SAP97 gene,^109^ p140Cap gene,^110^, ^111^, ^112^ Pten gene,^113^ and nSR100 gene.^114^

#### 3.2.4 Others

Previous omics studies have also revealed that physiological and metabolic abnormalities such as mitochondrial dysfunction, oxidation, and inflammation are associated with ASD. The mitochondrial deficiency is expected to explain the underlying damage mechanism in ASD. ASD were described as mitochondrial diseases and its potential mechanism was identified through phosphoproteomics.^115^ The alternated pathways in brain of autistic subjects were associated with energy metabolism, synaptic vesicle regulation as well as myelination.^116^ The change of mitochondrial function, energy metabolism, EIF2 signaling, immune functions, ubiquitination, and DNA repair were found in global proteomics of peripheral blood‐derived lymphoblasts with homozygous HERC2 variants.^117^ A transcriptome suggested that mitochondrial function, ribosome, and spliceosome components were down‐regulated in postmortem brain of ASD.^118^

A metabolic profiling of lymphoblastoid cells revealed a decreased tryptophan metabolism in ASD and showed a reduced generation of nicotinamide adenine dinucleotide (NADH), a critical energy carrier in mitochondria.^119^ The metabolic clusters containing lactate or pyruvate, succinate, α‐ketoglutarate, glycine, ornithine, and 4‐hydroxyproline highlighted potential dysregulation in amino acid and energy metabolism in ASD plasma.^120^ Importantly, a metabolomics in cerebrospinal fluid analysis from ASD showed that L‐cysteine, adenine, and dodecanoic acid were important metabolites for ASD.^121^ Additionally, amino acid and energy metabolism pathways were most disrupted in all neurodevelopment disorders.^121^ A previous study performed proteomics and metabolomics on amniotic fluids from pregnant woman with male fetuses and premutation in FMR1 gene. The result showed the mitochondrial dysfunction induced by the deficits in prenatal serine biosynthesis underlie.^122^ A wide range of aberrant mitochondria‐related pathways, including respiratory electron transport chain, cellular response to stress, regulation of neuron apoptotic process, and reactive oxygen species (ROS) metabolic process were triggered by SHANK3 mutation in mouse cortex.^123^ Untargeted metabolomics revealed that key metabolic mitochondrial/extramitochondrial pathways was up‐regulated in mecp2‐deficient mouse cortex.^124^ VPA‐induced alterations in metabolites of serum, urine, and brain cortex were associated with mitochondrial dysfunction metabolism and CNS disorders.^125^

A mechanistic modeling based on transcriptome suggested a direct link between inflammation and ASD in neurons.^118^ Notedly, the MIA is a one of the common environmental risk factors of ASD pathology during pregnancy.^126^, ^127^, ^128^ The adaptive immune pathway was enriched in maternal blood from mothers of children later diagnosed with ASD by transcriptome.^129^ Maternal inflammation with elevated kynurenine metabolites is related to the risk of abnormal brain development in ASD.^130^ Similarly, the increased paternal age at conception has been associated with ASD.^131^, ^132^

In the metabolic profile, prostaglandin D2, which is a type of inflammatory mediators was increased in plasma of young boys with ASD and implicated with neuroinflammation.^133^ In the liver of BTBR mouse model of autism, 12 differential metabolites suggested that bile acid‐mediated activation of LXRα might contribute to metabolic dysfunction of lipid and leukotriene D4 produced by the activation of 5‐LOX led to hepatic inflammation.^134^ In ASD children brain, abnormal levels of N‐acetyl‐compounds, glutamate glutamine, creatine phosphocreatine (Cr), or choline‐compounds (Cho) implicated that neuron or glial density, mitochondrial energetic metabolism, and/or inflammation contribute to ASD neuropathology.^135^ The consistent appearance of inflammation regulation in proteomics from Mecp2‐mutant mouse, cells generated from induced pluripotent stem cells (iPSC) in Rett syndrome (RTT), and RTT peripheral samples implied that it contributed to the destruction of the nervous system.^136^

**FIGURE 2:**
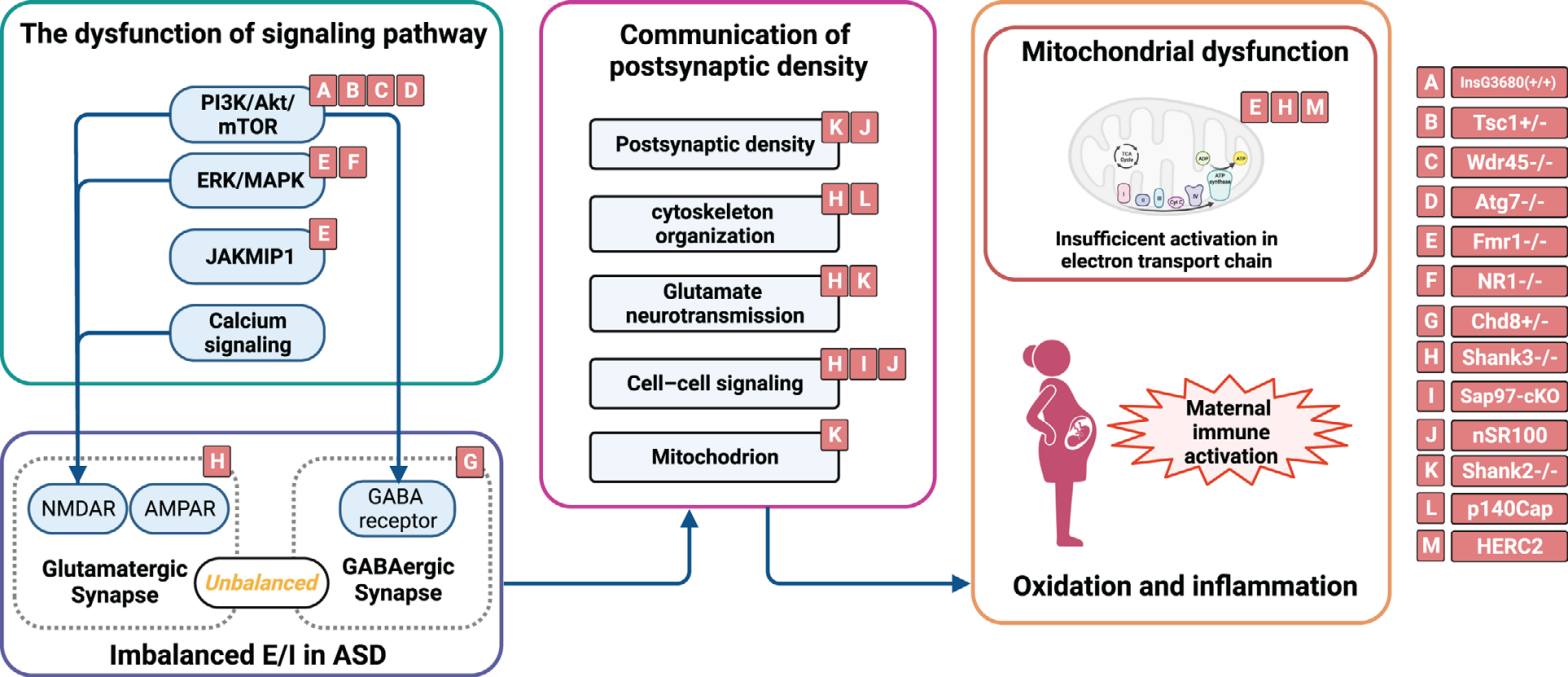
The graphical abstract of potential pathology of autism spectrum disorder (ASD) investigated by multi‐omics methods. The dysregulation of signaling pathways in neuron lead to abnormal balance between excitatory and inhibition. Many genes mutation influenced the postsynaptic density including the cytoskeleton organization, glutamate neurotransmission, cell–cell signaling, and mitochondrional function. The accumulation of negative effect further impacts the downstream of synapse. The maternal immune activation and mitochondrial dysregulation are associated with oxidation and inflammation. The red labels indicates that the genes are associated with the process in the studies. E/I, excitation and inhibition.

In summary, the above‐mentioned signal pathways play a significant role in the typical neurodevelopment process, and their dysfunction can lead to downstream alterations, such as an imbalance in excitatory and inhibitory synapses. This can result in the transmission of erroneous signals within neural circuits, may be caused by inflammation and reoxidation. The maintenance of stable neural communication is contingent upon the integration of synapse construction, such as PSD, and the provision of sustainable energy from mitochondria. As a result, a series of aberrant signaling molecules, excitatory and inhibitory imbalances, PSD, mitochondrial dysfunction, and inflammation ultimately lead to neural immaturity and damage in ASD pathology (Figure 2).

### 3.3 Studies on the pathogenesis of ASD in different genetic backgrounds

The search for “commonalities” among children with ASD has become a focus of current research and a breakthrough point.^137^, ^138^, ^139^, ^140^ ASD‐related syndromes with a clear genetic cause for the autism phenotype offer the best opportunity to elucidate the underlying mechanisms of ASD and to identify possible therapeutic targets^141^ and diagnostic markers.^142^ In recent years, there has been notable advancement in the identification of genes closely linked to ASD. These genes exhibited distinct molecular functions but may share biological pathways. In the context of known genes, the research on genes and pathological mechanisms, diagnostic markers, and even imaging is conducive to finding the commonality between different genes (Table 1).

**TABLE 1:** Studies on different genotypes of autism spectrum disorder (ASD).

No.	Author	Sample	Genotype	Method	Major finding
1	Ellegood et al. (2015)^143^	Mouse brain	15q11‐13, 16p11, AndR, BALB/c, BTBR, CNTNAP2, En2, FMR1, GTF2i, ITGβ3, Mecp2, NLGN3, NRXN1α, SLC6A4, SHANK3, XO	MRI	26 different mouse models were examined, the parieto‐temporal lobe, cerebellar cortex, frontal lobe, hypothalamus, and the striatum are the abnormal regions, unknown connections between Nrxn1α, En2, Fmr1, Nlgn3, BTBR, and Slc6A4 were identified.
2	Brown et al. (2018)^144^	Mouse FC, HC	CNTNAP2, FMR1, Shank3B, Shank3Δex4‐9, TSC2, Ube3a2xTG,	QMI	A unique set of disrupted interactions was displayed by each model, but synaptic activity‐related interactions were disrupted. Potential relationships among models and deficits in AKT signaling in Ube3a2xTG mice were confirmed.
3	Jin et al. (2020)^145^	Mouse CPN, CIN, AC, OC, MG	Adnp, Ank2, Arid1b, Ash1l, Asxl3, Chd2, Chd8, Cntnb1, Cul3, Ddx3x, Dscam, Dyrk1a, Fbxo11, Gatad2b, Kdm5b, Larp4b, Mbd5, Med13l, Mll1, Myst4, Pogz, Pten, Qrich1, Satb2, Scn2a1, Setd2, Setd5, Spen, Stard9, Syngap1, Tcf20, Tcf712, Tnrc6b, Upf3b, Wac	In vivo Perturb‐Seq	In vivo Perturb‐Seq can serve as a tool to reveal cell‐intrinsic functions at single‐cell resolution in complex tissues, which can be applied across diverse and tissues in the intact organism.
4	Carbonell et al. (2021)^146^	Mouse HC	Anks1b, BTBR, Cntnap2, Cacna1c, Fmr1, Pten, Shank3	TMT, SDS‐PAGE, LC–MS	Hippocampal synaptic proteomes from seven mouse models were identified, common altered cellular and molecular pathways at the synapse were also identified.
5	Zerbi et al. (2021)^147^	Mouse brain	16p11.2, BTBRT, CDKL5, CHD8, CNTNAP2, En2, FRM1.1, FRM1.2, Het, IL6, Mecp2, SGSH, SHANK3b, Syn2, TREM2	MRI	ASD‐associated etiologies cause a broad spectrum of connectional abnormalities, etiological variability is a key determinant of connectivity heterogeneity in ASD, identification of etiologically relevant connectivity subtypes could improve diagnostic label accuracy in the non‐syndromic ASD population.
6	Willsey et al. (2021)^148^	Xenpus tropicalis brain	ARID1B, ADNP, ANK2, CHD8, CHD2, DYRK1A, NRXN1, POGZ, SCN2A, SYNGAP1	LWI	Mutations lead to an increase in the ratio of neural progenitor cells to maturing neurons, systematic small molecule screening identifies that estrogen rescues the convergent phenotype and mitigate a broad range of ASD genetic risks.
7	Shen et al. (2022)^149^	Human blood	ASH1L, DDX3X, GIGYF2, NAA15, SCN2A	iTRAQ, LC–MS/MS, ELISA	The DEPs and differential metabolites of plasma could distinguish the cases form controls. Proteomic results highlighted complement, inflammation, immunity, mitochondrial dysfunction, proteasome, ubiquitin‐mediated proteolysis, and ER stress in the pathogenesis of ASD.
8	Paulsen et al. (2022)^24^	Human CC	ARID1B, CHD8, SUV420H1	IC, WB	Cell‐type‐specific neurodevelopmental abnormalities that are shared across ASD risk genes and are modulated by human genomic context were uncovered, convergence in the neurobiological basis of how different risk genes contribute to ASD pathology were found.
9	Pintacuda et al. (2023)^150^	Human brain excitatory iNs	ARID1B, ANK2, ADNP, CTNNB1, CHD8, DYRK1A, GIGYF1, MED13L, PTEN, SCN2A, SYNGAP1, SHANK3, TLK2	WB, LC–MS/MS	The ASD‐linked brain‐specific isoform of ANK2 is important for its interactions with synaptic proteins and to characterize a PTEN–ANKAP8L interaction that influences neuronal growth, the IGF2BP1–3 complex emerged as a convergent point in the network that may regulate a transcriptional circuit of ASD‐associated genes.
10	Carbonell et al. (2023)^146^	Mouse HC	Anks1b, BTBR, Cntnap2, Fmr1, Pten	TMT, LC–MS	Changes in oxidative phosphorylation and Rho family small GTPase signaling were revealed, the ANKS1B model displays altered Rac1 activity counter to that observed in other models was confirmed.
11	Mendes et al. (2023)^151^	Zebrafish brain	CHD8, CNTNAP2, CUL3, DYRK1A, GRIN2B, KATNAL2, KDM5B, SCN2A, TBR1, POGZ	IC, CI, RNA‐Seq	A global increase in microglia resulting from ASD gene loss of function in select mutants, implicates neuroimmune dysfunction as a key pathway relevant to ASD biology.

Abbreviations: AC, astrocytes; CC, cerebral cortex; CI, confocal imaging; CIN, cortical inhibitory neurons; CPN, cortical projection neurons; ELISA, Enzyme‐linked immuno sorbent assay; ER, Endoplasmic reticulum; FC, frontal cortex; HC, hippocampus; IC, immunohistochemistry; iNs, induced neurons; LC–MS/MS, liquid chromatography–tandem mass spectrometry; LWI, large‐scale whole‐brain imaging; MG, microglia; MRI, magnetic resonance imaging; OC, oligodendrocytes; QMI, quantitative multiplex co‐immunoprecipitation; SDS‐PAGE, sodium dodecylsulfate polyacrylamide gel electrophoresis;TMT, tandem mass tag system; WB, western blot.

A recent study of iPSC‐derived “brain‐like organs” from children carrying three different ASD risk genes showed that although each gene acts through a unique underlying molecular mechanism, they have similar overall defects that affect similar aspects of neurogenesis and the same type of neurons.^24^ Using iPSC, Pintacuda et al. constructed protein–protein interaction networks among 13 ASD‐related genes in human excitatory neurons, revealing the neuron‐specific biology associated with ASD.^150^ Three animal experiments with known genetic backgrounds suggest that synapses play a key role in the pathogenesis of ASD.^144^, ^146^ Among them, Jordan and coworkers compared the synaptic proteomes of five mouse models of autism revealing convergent molecular similarities, including defects in oxidative phosphorylation and Rho GTPase signaling.^146^ They also compared synaptic proteomes of seven mouse models of autism revealing molecular subtypes and defects in Rho GTPase signaling.^146^ Another study investigated seven animal models of ASD and showed that there is great heterogeneity between models. However, high‐dimensional measurements of synaptic protein networks may allow a promising avenue for subtype differentiation of ASD with common molecular pathology. Notably, this approach demonstrated convergence between the glutamate synapse protein interaction networks of the VPA and TSC2 mouse models, both converging on a putative “mTOR” cluster.^144^

Similarly, a previous study identified distinct and overlapping phenotypes at the level of behavior, brain structure and circuitry by analyzing the function of 10 autism genes in zebrafish. The study observed that the forebrain contributed most to brain size differences between ASD genes, that brain activity phenotypes were concentrated in regions involved in sensory‐motor control, that dopaminergic and microglia abnormalities were the confluence of two genes (SCN2A and DYRK1A), and implied that neuroimmune dysfunction was associated with autism biology.^151^ In addition, Willsey et al. employed parallel in vivo analyses and systems biology approaches to examine 10 genes linked to ASD by utilizing tropical African clawed toads. The results suggested that cortical neurogenesis served as a convergence vulnerability site in ASDs. Moreover, estrogen is a restorative factor for several different autism genes and they revealed a conserved role for estrogen in inhibiting sonic Hedgehog signaling.^152^

In vivo Perturb‐Seq technology based on CRISPR‐Cas9 and single‐cell RNA sequencing technology developed a high‐throughput genetic screening method to study the function of numerous genes in complex tissues at single‐cell resolution. Recently, Zhang and coworkers applied this method to analyze the effects of 35 ASD/ND risk genes on brain development in mice. The authors identified cell type specific and evolutionarily conserved gene modules from neuronal and glial cell categories.^145^

These studies exemplify the examination of genetic heterogeneity in ASD by conducting studies of common features of ASD and controls based on known genetic backgrounds. The findings suggested that ASD‐associated susceptibility genes ultimately converge on common signaling pathways and that these convergence sites are key to understand ASD pathology. Therefore, categorizing genes based on shared biology despite their heterogeneity might represent a path toward precision medicine in ASD, bridging the gap between gene discovery and actionable biological mechanisms.^151^

Moreover, similar results have been obtained in imaging studies under different genetic backgrounds. Functional magnetic resonance imaging analysis of 16 mouse mutants with ASD‐related mutations identified brain connectivity subtypes among the mutants despite the presence of distinct phenotypes.^147^ Likewise, although mouse mutants with 26 ASD genes exhibited heterogeneous neuroanatomical phenotypes, clustering of these mutants by shared features allowed identification of gene subgroups.^143^

Overall, these studies suggest that conducting research on the convergent mechanisms among ASD‐related genes and elucidating the shared pathways could provide information to unravel the mechanisms of ASD and explore potential therapeutic targets and diagnostic biomarkers.

### 3.4 Common mechanisms associated with ASD and its comorbidities

The comorbidities in most children with ASD is a notable attribute, contributing to its diverse and intricate nature.^153^ Thus, investigating common mechanisms between ASD and comorbidities, as well as the specific genes and mechanisms that lead to their respective occurrence, is a topic of interest in the field of ASD research, and its study contributes to the diagnosis and treatment of ASD. Previous studies have shown some common mechanisms between these comorbidities and ASD.^153^ For example, recent studies have highlighted points of convergence between ASD and neurodevelopmental disorders (NDD) genes.^154^ Chromosomal microarray and sequencing studies have identified significant genetic overlap between ASD and other NDD and neurological disorders, including ID, epilepsy, and schizophrenia.^155^, ^156^ Two meta‐analyses of genome‐wide associations have also shown that ASD shares a common genetic background in neuropsychiatric disorders.^157^, ^158^ Genes involved in synaptic structure and function are implicated in a variety of disorders, including schizophrenia, ASDs, and other NDDs.^159^, ^160^, ^161^ The gene discovery can help to distinguish this complexity by analyzing the genetic structure and risk gene associations of different subtypes or comorbidities. In addition, several environmental factors have been found to be associated with ASD and its comorbidities, such as MIA in the prenatal environment, stress, drug exposure, and malnutrition,^126^, ^127^, ^162^, ^163^, ^164^ as well as gastrointestinal dysfunction and disruption of intestinal flora.^165^, ^166^, ^167^ These studies suggest that although the heterogeneity of ASD is complicated by the occurrence of comorbidities, common mechanisms may still be found between ASD and its comorbidities.

### 3.5 Mechanisms associated with important physiological and metabolic abnormalities

As mentioned above, immune dysregulation, inflammation, oxidative stress, and mitochondrial dysfunction are closely associated with ASD and are important physiological and metabolic abnormalities in ASD.^128^, ^138^, ^168^, ^169^, ^170^ They may be the intersection of genetic and environmental factors and contribute to ASD.

Immunity and neuroinflammation play a key role in the development of ASD.^171^, ^172^, ^173^ Immune dysfunction in ASD involves a network of interactions between several cell types from the innate and adaptive immune response. Multiple immune factors mediate the effects of CNS function. Some cytokines inhibit neurogenesis and promote neuronal death, whereas others promote the growth and proliferation of neurons and oligodendrocytes. Complement proteins and microglia can be involved in synaptic scaling and pruning, while brain‐reactive autoantibodies can alter neuronal development or function.^172^ Active microglia and astrocytes have been observed in the brains of ASD. Activation of microglia in different brain regions was observed, including an increase in cell number or cell density, morphological changes, and phenotypic alterations.^174^ Activation of microglia releases inflammatory cytokines and chemokines such as interleukin (IL)‐6, IL‐12, IL‐β, and tumor necrosis factor‐alpha (TNF‐α). Excessive induction of nitric oxide synthase (NOS) and ROS affects synaptic plasticity and produces behavioral abnormalities associated with ASD.^175^

Oxidative stress is associated with mitochondrial dysfunction. Decreased endogenous antioxidant capacity, particularly reduced total glutathione (tGSH) levels and altered glutathione peroxidase (GPx), superoxide dismutase, and catalase activities, have been reported in ASD, which is consistent with elevated oxidative stress indicators in children with ASD.^176^ The prevalence of mitochondrial disease in ASD is 4%−5%, which is significantly higher than in the general population (about 0.01%).^177^, ^178^ Mitochondrial abnormalities such as increased hydrogen peroxide, decreased NADH, and mitochondrial DNA over‐replication have been observed in lymphocytes isolated from subjects with ASD.^179^ Mitochondria produce adenosine triphosphate (ATP). Reduced ATP production and elevated levels of lactate and pyruvate in individuals with ASD may indicate mitochondrial dysfunction in autism.

These physiologic and pathologic processes interact with each other, and multiple mechanisms are interrelated.^128^, ^170^, ^180^, ^181^ Oxidative stress can lead to mitochondrial dysfunction, and abnormal mitochondrial function leads to increased ROS metabolism and oxidative stress, creating a vicious cycle. The association between gut flora and MIA is also reflected in the pathogenesis of ASD.^128^, ^180^ MIA induces an immune response in pregnant women, leading to further inflammation and oxidative stress, as well as mitochondrial dysfunction in the placenta and fetal brain. These negative factors lead to neurodevelopmental deficits in the developing fetal brain, which subsequently lead to symptoms of behavioral disorders in the offspring.^128^ In summary, accumulating research into these common pathophysiologic mechanisms will enhance our comprehension of ASD diagnosis and treatment, while provide insight into general or subgroup‐specific processes that may contribute to the development of ASD and other psychiatric disorders.^168^

### 3.6 Pathological mechanisms of ASD associated with gut microbiota

**FIGURE 3:**
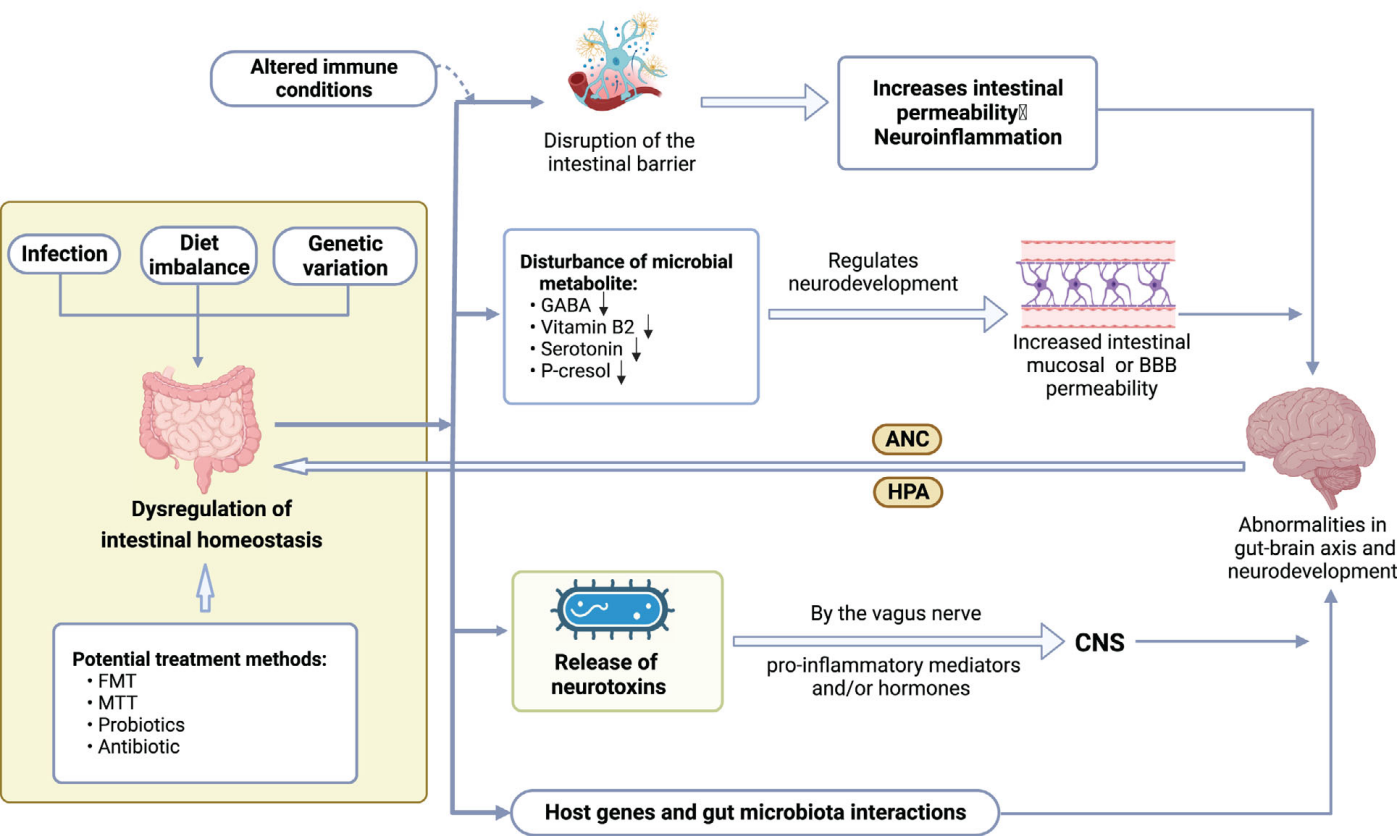
Potential mechanisms of gut microbiota imbalance and autism spectrum disorder (ASD) occurrence. The gut flora and brain can interact through immune, metabolic, and gut nervous system pathways and ultimately leading to abnormalities in gut brain axis and neural development. Gut flora alternation causes metabolism changes and its dysbiosis linked to greater intestinal mucosa and blood–brain barrier (BBB) permeability. Feedback regulation exists in gene expression, dietary preference, and gut flora. ANC, autonomic nervous system; CNS, central nervous system; FMT, fecal microbiota transplantation; HPA, hypothalamic–pituitary–adrenal; MTT, microbiota transfer therapy.

A series of studies have reported significant differences in the composition of gut microbiota between ASD cases and healthy controls (Figure 3). Changes in gut microbiota cause changes in metabolism. Several animal experiments have demonstrated an association between ASD and gut microbiota. Transplantation of gut flora from the individuals with ASD into germ‐free mice leads to autism‐like symptoms in the mice, which may be related to the regulation of tryptophan and 5‐hydroxytryptaminergic synaptic metabolism^182^ or it may lead to alterations in neuroactive metabolites.^183^ It has also been found that changes in the gut microbiota of children with ASD affect glutathione (GSH) synthesis^184^ and degradation of organic toxins, lacking biosynthetic pathways for several neurotransmitters^185^ or vitamins.^186^ In addition, some bacterial metabolites may contribute to the development of autism‐like behaviors, such as elevated acetaminophen sulfate levels.^187^, ^188^ The presence of gut dysbiosis has also been linked to heightened permeability of the intestinal mucosa or the blood–brain barrier. For example, abnormal metabolism of some short‐chain fatty acids (SCFAs) affected tight junction proteins associated with blood–brain barrier permeability.^189^ The neurotoxins are released by a variety of harmful bacteria that are delivered via the enteric vagus nerve to the CNS.^182^ The permitting pro‐inflammatory mediators and/or hormones enter the circulation and to be transported from bloodstream to the brain, where they may ultimately affected the CNS neurodevelopment and/or function.^182^

Studies on Cntnap2 double knockout^190^ and CHD8 single knockout autism model mice^191^ have shown that the combined effects of host genes and gut flora in interacting with each other lead to behavioral abnormalities in autism. Genetic factors and dietary habits can alter the composition of the gut microbiota, while imbalances in the gut microbiota can also trigger aberrant gene expression and influence dietary preferences.^192^ Moreover, the nervous system can act on the gastrointestinal tract and its microbiota through specific pathways (e.g., the autonomic nervous system axis and the hypothalamic–pituitary–adrenal axis) to regulate intestinal motility and secretion, and to influence gut microbial composition and function.

Furthermore, studies of gut microbiota have also revealed that pathological inflammation of ASD occurs not only in the CNS and periphery, but also in the gut. The damaged, inflamed, and permeable epithelia are the predominant routes utilized by commensal bacteria to migrate to the bloodstream.^193^ The microbial metabolites are likely the most significant contributors to systemic inflammation and subsequent neuroinflammation.^193^, ^194^ The occurrence of abnormal oxidation or unsuitable activation of immune led to subsequent inflammation and neuroinflammation in CNS, periphery, and gut of ASD.

Taken together, dysbiosis of the gut microbiota may be an important contributor to ASD, leading to disruptions in gut–brain axis connectivity and neurodevelopment caused by bacterial metabolites, the enteric nervous system, and the systemic immune system. An in‐depth exploration of the possible molecular mechanisms by which gut microbes influence behavioral changes in ASD offers great potential for intervention, diagnosis, and therapeutic evaluation in ASD. Notably, to date, the relationship between the gut microbiota and autism symptom severity is difficult to determine, and no specific bacterial group could be identified as being solely responsible.^195^

## 4 STUDY ON DIAGNOSTIC BIOMARKERS OF ASD

A widely accepted consensus in clinical practice is that timely identification and diagnosis play a crucial role in facilitating early intervention and prognostic outcomes. To achieve this goal, the American Academy of Pediatrics recommends that all children should be screened for autism for the first time at 9 months of age and at routine developmental monitoring centers at 18, 24, and 30 months of age.^196^ In China, there are similar consensus or norms.^197^ Therefore, it is necessary to identify early behavioral features of ASD that can be used for early diagnosis. Moreover, there is a need to investigate biomarkers for objective diagnosis. Measurable laboratory biomarkers may be an opportunity to identify risks that not only provide an earlier and more reliable diagnosis, but further differentiate the autism spectrum based on common pathophysiological features, allowing for individualized treatment and response monitoring, and increasing the chances of success of future drug development programs.^198^ To date, some consensus has raised on the early behavioral features of ASD.^28^, ^196^ Although progress has been made in the study of diagnostic markers, most biomarkers have not yet been validated and further research is required.

A recent study conducted a systematic review of diagnostic molecular markers for ASD.^199^ The majority of these markers are measured peripherally via blood, and although there is considerable variation between and within individual biomarkers, two major groups are apparent, one consisting of cytokines and growth factors (e.g., IL‐6, brain‐derived neurotrophic factor) and the other consisting of amino acids, neurotransmitters (e.g., cysteine, serotonin, GABA), and hormones (e.g., vitamin D). In between these two groups are molecules related to reduction/oxidation (redox), including GSH, which is the most frequently detected molecule. Most papers also report an association between molecular markers and ASD diagnostic status.

In this section, we provide an overview in terms of non‐targeted omics and targeted research of ASD diagnostic markers, as well as research on diagnostic markers associated with important physiological and metabolic abnormalities of ASD, and gut microbiota.

### 4.1 The identification of potential biomarkers by non‐targeted omics

Genetic testing, proteomics, and metabolomics were employed in previous study to screen a number of genes, proteins, peptides, and metabolites that have the potential to be diagnostic markers for ASD.^18^ Protein and metabolite‐based tests provided the highest diagnostic accuracy for ASD, which combined with multiple features may further improve diagnostic accuracy.^200^

There have been several reports and reviews on the proteomics of ASD protein diagnostic markers, mainly including blood, urine, and saliva studies. Overall, candidate proteins obtained from proteomic studies have little or no reproducibility in independent cohorts.^201^ However, bioinformatics analysis showed that the majority of proteins in different studies were associated with complement and coagulation cascades, focal adhesion, platelet activation, vitamin digestion and absorption, immune response, inflammatory response, cholesterol metabolism, lipid metabolism, oxidative stress, and energy metabolism. These mechanisms are evidently prevalent in individuals with ASD, thus indicating a convergence of protein‐associated mechanisms that hold promise as potential diagnostic markers.^201^, ^202^, ^203^, ^204^, ^205^

Metabolism‐based analyses have the advantage of being sensitive to the interactions between genomic, gut microbiome, dietary, and environmental factors. The metabolite differences between disease and normal states has received increasing attention in recent years. Studies of blood and urine metabolomics in children with autism versus controls have shown that although fewer metabolisms show consistent changes across studies, the mechanisms by which they are associated are convergent and correlate with common pathogenesis and pathophysiological changes in ASD. Changes in blood metabolites are mainly associated with mitochondrial dysfunction, oxidative stress, fatty acid metabolism, energy metabolism, cholesterol metabolism, neurotransmitters, and mammalian–microbial co‐metabolism pathway.^18^, ^206^ Most of the changes in urinary metabolites are related to amino acid metabolism, energy metabolism, oxidative stress, intestinal flora, and neurotransmission. The metabolism of some amino acids (e.g., tryptophan and branched‐chain amino acids) and neurotransmitters (e.g., glutamate, ROS, and lipids) may play an important role in the pathogenesis of ASD.^18^, ^206^

A recent study analyzed blood and urine metabolites from the same group of children with autism and found decreased urinary taurine and catechol levels and increased plasma taurine and catechol levels.^207^ Another urine metabolomics study in twins found that phenylpyruvate and taurine were elevated in the autistic group, while carnitine was decreased, and arginine and proline metabolic pathways were enriched. In twins, there was a significant positive correlation between indole‐3‐acetate and autistic traits.^208^ In addition, in some recent omics studies,^209^, ^210^, ^211^, ^212^ machine learning methods have been used to screen diagnostic markers from omics data.

The combined multi‐omics approach has been reported in several studies of diagnostic markers for ASD.^142^, ^213^, ^214^ For example, using metabolomic and transcriptomic approaches, Dai et al. revealed that blood uric acid levels were significantly lower in children with ASD and the expression levels of some genes related to purine metabolism differed between children with ASD and controls.^213^ Integrated proteome and metabolome analysis, another study found that six signaling pathways were significantly enriched in ASD, three of which were correlated with impaired neuroinflammation (GSH metabolism, metabolism of xenobiotics by cytochrome P450, and transendothelial migration of leukocyte).^214^ Although further validation is needed, in combination with proteomic and metabolomic data, a previous study suggests that glycerophospholipid metabolism and N‐glycan biosynthesis may play a key role in the pathogenesis of ASD.^142^

Moreover, to explore the effect of ASD gene heterogeneity on the study and application of diagnostic markers, Shen et al. preliminarily detected five children with ASD carrying risk genes for ASD from 126 cases through gene‐targeted testing, proteomic, and metabolomics in plasma and peripheral blood mononuclear cells (PBMCs) compared to healthy controls.^142^ The results showed that although the children with ASD differed in their expression patterns of total proteins and metabolites, the differential proteins and metabolites identified were still able to distinguish cases from controls well, and the mechanisms of association were consistent with those reported in previous studies.^18^ Based on this, they added the group of children clinically diagnosed with ASD but not detected as carrying risk genes to further the study and obtained similar conclusions.^215^ These findings support that, despite the presence of genetic heterogeneity, it is possible to identify markers for diagnosis among children with different genetic backgrounds.

### 4.2 Targeted research and application of diagnostic markers

The targeted validation and detection of diagnostic markers, especially using some high‐throughput methods (e.g., targeted proteomics, metabolomics), is convenient and important. This is primarily due to the utilization of multiple markers in the combined diagnosis of multifactorial diseases, which typically results in enhanced diagnostic accuracy and specificity compared to single diagnostic marker. Here, we focus on targeted proteomics and metabolomics studies. However, in reality, any study that addresses the common pathophysiological mechanisms associated with ASD is also a targeted study, such as studies that have selected a panel of cytokines for peripheral blood testing based on literature reports.^216^, ^217^ Studies targeting a particular class of biomarkers related to oxidative stress, mitochondria, gut microbiota, etc., are also in line with this idea. They are reviewed in Sections 4.3 and 4.4. Indeed, genetic testing with a panel consisting of known ASD‐related genes should also be included.^161^, ^218^, ^219^

#### 4.2.1 Targeted proteomics research

Applying targeted proteomics multiple reaction monitoring technology, we have previously performed targeting studies on the proteins of ASD plasma complement and coagulation cascades, and combined with machine learning methods, we obtained a set of 12 differential protein combinations with diagnostic potential.^212^ The complement system composed of more than 40 proteins served as an important component of the human immune system. The expression of complement or complement and coagulation cascade‐related proteins has been frequently reported alteration in the peripheral blood of ASD since the first proteomic studies on peripheral blood in ASD,^18^, ^142^, ^211^, ^220^, ^221^, ^222^ while changes in the brain have also been reported.^221^, ^222^ The association of complement with neuropsychiatric disorders has recently attracted attention.^221^, ^222^ The correlation between alterations of complement proteins in brain and periphery of children with ASD remains unclear, and the underlying mechanisms are not comprehensively understood, thus necessitating further research.

#### 4.2.2 Targeted metabolomics studies

Metabolomics is capable of identifying biochemical imbalances that are frequently present in children with ASD, primarily involving amino acids, reactive oxidative stress, neurotransmitters, and the microbial–gut–brain axis,^206^, ^223^ and their changes further support the association of these mechanisms with ASD. Studies on the targeted metabolomics of ASD are progressing rapidly, including those on the targeted metabolomics of body fluids such as blood and urine. We have summarized them in Table 2.

**TABLE 2:** Research on potential biomarkers of autism spectrum disorder (ASD) based on targeted metabolomics.

No.	Author	Sample	Method	Related metabolites	Metabolic process involved
1	West et al. (2014)^224^	Blood	GC–MS, LC–HRMS	Decreased^a^: homocitrulline, citric acid, lactic acid, heptadecanoic acid, myristic acid Increased^a^: aspartic acid, serine, glutamic acid, glutaric acid, soleucine acid, 2‐hydroxyvaleric, 3‐aminoisobutyric acid, 5‐hydroxynorvaline	Mitochondrial dysfunction, abnormal gut microbiome metabolism
2	Anwar et al. (2018)^225^	Blood	LC–MS/MS	Decreased^b^: FL, G‐H1, NFK Increased^b^: CMA, AASA, GSA, arginine, glutamic	Abnormal protein glycosylation, protein oxidative metabolism
3	Delaye et al. (2018)^226^	Blood	Ion exchange chromatography	Decreased^b^: glutamate, serine, ornithine, proline	Glutamate neurotransmission, gastrointestinal abnormalities
4	Lv et al. (2018)^227^	Blood	MS/MS	Decreased^a^: free carnitine, glutaricyl carnitine, octyl carnitine, 24 carbonyl carnitine, carnosyl carnitine	Mitochondrial dysfunction, abnormal fatty acid metabolism
5	Smith et al. (2019)^228^	Blood	LC–MS/MS, MRM	Decreased^a^: leucine, isoleucine, valine Increased^a^: glutamine, glycine, ornithine	Protein synthesis, neurotransmission, AA/BCAA metabolism
6	Brister et al. (2022)^229^	Blood	LC–MS/MS	Decreased^b^: Nε‐fructosyl‐lysine Increased^b^: Nω‐carboxymethylarginine, Nε‐(1‐carboxyethyl) lysine, glutamic semialdehyde, 3‐nitrotyrosineα‐aminoadipic semialdehyde	Energy metabolism, amino acid neurotransmitter metabolism, branched‐chain amino acid metabolism, nicotinamide metabolism, aminoacyl tRNA biosynthesis
7	Shen et al. (2022)^149^	Blood	LC–MS/MS	Decreased^b^: L‐glutamate, pyridoxamine, O‐phospho‐4‐hydroxy‐L‐threonine, L‐aspartate, 4‐pyridoxate, phosphatidylethanolamine, 2‐oxoglutaramate Increased^b^: L‐glutamine, creatineacetylglycine, serylserine, 1‐acyl‐sn‐glycero3phosphocholine, ornithine, phosphatidylserine	Mitochondrial dysfunction, oxidative stress, energy metabolism, amino acid, vitamin, lipid metabolism
8	Kaluzna‐ Czaplinska et al. (2010)^230^	Urine	GC–MS	Increased^a^: urine homovanillic acid, vanilla mandelic acid	Neurotransmitter metabolism, visual perception/memory, repetitive behavior, emotional disorders
9	Mavel et al. (2013)^231^	Urine	^1^H‐^13^C NMR	Decreased^b^: creatine, 3‐methylhistidine Increased^b^: glycine, taurine, succinate, β‐alanine	Taurine and succinic acid
10	Emond et al. (2013)^232^	Urine	GC–MS	Decreased^b^: 1H‐indole‐3‐acetate, phosphate, palmitate, stearate, 3‐methyladipate, hippurate, vanillylhydracrylate, 4‐hydroxyphenyl‐2‐hydroxyacetate, 3‐hydroxyphenylacetate Increased^b^: succinate, glycolate	Intestinal bacteria microbial pathways
11	Nadal‐ Desbarats et al. (2014)^233^	Urine	^1^H‐NMR, ^1^H‐^13^C HSQC‐NMR	Decreased^b^: glutamate, creatine, 3‐methylhistidine Increased^b^: succinate	Energy metabolism disorder, mitochondrial dysfunction, amino acid metabolism of gut microbiota
12	Liu et al. (2019)^234^	Urine	LC–MS/MS	Decreased^a^: Lys, Thr, Car, Pro, EtN, Hcy, Aad, Cit, Ans, 5Ava, Asp Increased^a^: MetS, Harg, 3MHis, Cr, Arg, 5HT, Hyp	Oxidative stress, abnormal ornithine cycle, abnormal lysine metabolism, abnormal 5HT metabolism, E/I balance

Abbreviations: 3MHis, 3‐methyl‐histidine; 5Ava, 5‐aminovaleric acid; 5HT, 5‐hydroxytryptamine; Aad, α‐aminoadipic acid; AA/BCAA, amino acids/branched‐chain amino acid; AASA, α‐aminoadipic semialdehyde; Ans, anserine; Arg, arginine; Asp, aspartic acid; Car, carnosine; Cit, citrulline; CMA, Nω‐carboxymethylarginine; Cr, creatinine; E/I, excitation and inhibition; EtN, ethanolamine; FL, Nε‐fructosyl‐lysine; GC–MS, gas chromatography–mass spectrometry; G‐H1, hydroimidazol one; GSA, glutamic semialdehyde; Harg, homoarginine; Hcy, homocysteine; HSQC‐NMR, heteronuclear singular quantum correlation‐nuclear magnetic resonance; Hyp, 4‐hydroxyproline; LC–HRMS, liquid chromatography–tandem high‐resolution mass spectrometry; LC–MS/MS, liquid chromatograph–tandem mass spectrometry; Lys, lysine; MetS, methionine sulfoxide; MRM, multiple reaction monitoring; NFK, N‐formylkynurenine; Pro, proline; TD, typically developing; Thr, threonine.

^a^ ASD compared to TD.

^b^ ASD compared to Ctrl.

At present, targeted detection of metabolites altered in blood include amino acids (tyrosine, tryptophan, arginine, proline, methionine, cysteine, and taurine), lipids (phospholipids, sphingolipids, and fatty acids), and metabolites in the urea cycle and xenobiotics metabolism.^142^, ^235^ The metabolites associated with branched‐chain amino acid (BCAA) metabolism,^236^ fatty acid metabolism (free carnitine, short‐ and long‐chain acylcarnitine),^227^ tricarboxylic acid (TCA) cycle, fatty acids, oxidative phosphorylation, mitochondrial dysfunction, gut microbiome metabolism,^142^, ^237^ and neurotransmitter metabolism^238^ in the plasma of ASD are also involved.

Similarly, in targeted metabolomics studies of urine, previous studies have targeted the abnormalities of reactive oxidative stress, gut bacteria metabolism,^239^ amino acid (tyrosine, tryptophan, arginine, proline, methionine, cysteine, and taurine), lipid (phospholipid, sphingolipid, and fatty acid), urea cycle, xenobiotics metabolism,^239^, ^240^ TCA cycle, and glutamate metabolism^240^ in urine of ASD. Additional studies have also observed abnormalities of ornithine (urea) cycle, methionine, lysine, reactive oxidative stress, and tryptophan–serotonin metabolism in urine of children with ASD.^239^ Of interest, a prior study applied a targeted metabolomics approach to examine markers of oxidative stress and gut microbiota dysbiosis reported in previous studies and determined that levels of methylguanidine and n‐acetylarginine, which are associated with oxidative stress, and the gut bacterial metabolites indolol sulfate and indole‐3‐acetic acid were elevated in the urine of children with ASD.^241^

### 4.3 Study of biomarkers associated with important physiological and metabolic abnormalities in ASD

#### 4.3.1 Biomarkers associated with immunity/inflammation

The mounting evidence of altered central and peripheral immune system function supports to the notion that a subgroup of ASD may exhibited some form of immune system dysregulation.^242^ The levels of different cytokines in the peripheral blood of ASD have been extensively investigated, and several meta‐analyses have reviewed the relationship.^243^, ^244^, ^245^, ^246^ A systematic review and meta‐analysis showed that the pro‐inflammatory cytokines interferon (IFN)‐γ, IL‐1β, and IL‐6 were elevated in blood of children with ASD, while the anti‐inflammatory cytokine transforming growth factor‐β1 was decreased. Levels of several chemokines associated with recruitment of inflammatory cells, including eotaxin, IL‐8, and monocyte chemotactic protein‐1 (MCP‐1), were elevated. Another meta‐analysis showed that individuals with autism had lower levels of the anti‐inflammatory cytokines IL‐10 and IL‐1Ra, and higher concentrations of the pro‐inflammatory cytokines IFN‐γ, IL‐1β, IL‐6, and TNF‐α than controls.^245^ Also, meta‐regression analyses point to the interaction of latitude, age, and gender with peripheral alterations of associated pro‐inflammatory cytokines.^244^ A recent meta‐analysis found that the levels of peripheral IL‐6, IL‐1b, IL‐12p70, MIF, eotaxin‐1, MCP‐1, IL‐8, IL‐7, IL‐2, IL‐12, TNF‐α, IL‐17, and IL‐4 were significantly changed in ASD compared with controls. These findings reinforce the clinical evidence that ASD is associated with an abnormal inflammatory response. These cytokines may be a series of potential biomarkers in the peripheral blood of ASD.^246^ Besides, previous studies have reported that levels of some pro‐ and anti‐inflammatory cytokines and chemokines are associated with severity of abnormal behavior and impaired developmental and adaptive functioning.^247^, ^248^, ^249^ For example, IL‐6 has been extensively studied and its levels are elevated in ASD and correlate with severity.^199^ Indeed, cytokine changes have also been reported in postmortem brain tissue^250^, ^251^ and PBMCs.^248^ The changes in mRNA expression of some cytokines were found in whole blood from subjects with ASD.^252^

#### 4.3.2 Oxidative stress‐related biomarkers

In terms of markers associated with oxidative stress, a recent meta‐analysis showed that blood levels of oxidized glutathione (GSSG), malondialdehyde, homocysteine, S‐adenosylhomocysteine, nitric oxide, and copper were higher in children with ASD than in healthy controls, whereas GSH, tGSH, GSH/GSSG, tGSH/GSSG, methionine, cysteine, vitamin B9, vitamin D, vitamin B12, vitamin E, S‐adenosylmethionine/S‐adenosylhomocysteine, and calcium concentrations were decreased.^253^ Given the consistent and large effective size, GSH metabolism biomarkers have the potential to inform early diagnosis of ASD.^253^

Biomarkers of oxidative stress associated with ASD have recently been reviewed.^254^ GSH is an important antioxidant in the human body, it is converted to GSSG by GPx and reduced back to GSH by GSH reductase. Elevated levels of oxidative stress in ASD cause increased GSH depletion, which disrupts the dynamic balance between GSH and GSSG. The increased GSH/GSSG ratio is consistent with various pertinent studies, indicating that its efficacy as a reliable indicator of oxidative stress.^254^ In addition, blood levels of vitamin B9 and B12 were significantly lower in children with autism than in controls,^253^, ^255^, ^256^ and this deficiency resulted in decreased homocysteine remethylation and increased homocysteine levels. Vitamin B12 deficiency may lead to hypomethylation and affect brain development.^257^ Vitamin deficiencies in children with ASD may be due to poor nutrition, poor digestion, and absorption, or dysbiosis of the intestinal flora.^95^, ^254^ These results clarified blood oxidative stress profile in children with ASD, strengthening clinical evidence of increased oxidative stress implicating in pathogenesis of ASD.

#### 4.3.3 Mitochondria‐related diagnostic markers

A meta‐analysis showed that the regulation of mitochondrial biomarkers (including lactate, pyruvate, carnitine, and ubiquinone) was decreased in ASD, and that some of these markers correlated with ASD severity.^177^

### 4.4 Biomarkers associated with gut microbiota

Changes in the gut microbiota and metabolites may lead to changes in metabolites in blood and urine, providing an opportunity to develop diagnostic tests for early detection of ASD. For example, studies have shown that combining Veillonella and Enterobacteriaceae and 17 bacterial metabolic functions to create diagnostic models can effectively differentiate between ASD and healthy children.^258^ Several studies have shown that high levels of p‐cresol are detected in stool, blood, and urine of children with ASD.^18^, ^224^, ^259^, ^260^, ^261^, ^262^ Of interest, p‐cresol is only produced in the gastrointestinal tract and correlates with autistic behavior and ASD severity.^263^ In addition, other gut microbial metabolites including SCFAs, free amino acids, indoles, and lipopolysaccharides, have been detected in the blood and urine from children with ASD.^263^, ^264^ The analysis of gut microbes and the detection of microbial‐derived metabolites in stool, as well as the detection of gut microbial‐derived metabolites in blood and urine, may provide an alternative method for the early diagnosis of ASD and is worthy of initiating research (Table S2).

Overall, current research on diagnostic biomarkers for ASD suggests that despite the presence of heterogeneity in ASD, it is still possible to find diagnostic biomarkers. The mechanisms involved in the candidate diagnostic biomarkers identified in the existing studies are convergent. In the high‐throughput screening stage, there is still a lack of unified research methods, especially unified experimental conditions, and some studies need to overcome the shortage of small sample sizes. The targeted detection methods is beneficial for the practical application and translation of potential diagnostic biomarkers. It may be a panel composed of biomarkers involved in different mechanisms, or biomarkers related to a certain type of important physiological and metabolic changes.

#### 4.4.1 Intervention and treatment of ASD

**FIGURE 4:**
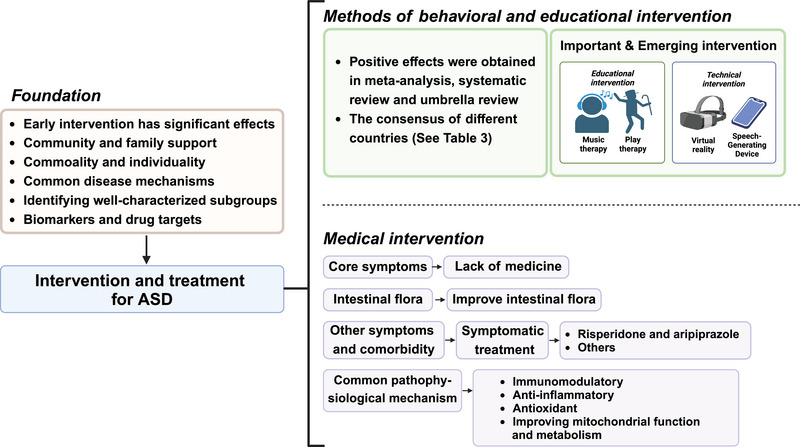
The summary of intervention therapy in autism spectrum disorder (ASD). Interventions for ASD mainly include behavioral and educational interventions, and we provide an overview of recent meta‐analyses, reviews, and consensus on them, as well as other important and emerging interventions. In terms of pharmacologic interventions, there are still no medications that target the core symptoms, and drug treatment is mainly for other abnormal symptoms or neuropsychiatric comorbidities of ASD. Treatments for common pathophysiology and gut flora are under investigation. Overall, early intervention has a significant effect, with community and family support being important. Given the characteristics of ASD, intervention and treatment need to take into account both commonalities and individuality. Finding common disease mechanisms and identifying well‐characterized subgroups will provide the basis for disease diagnosis and treatment, and disease markers and drug targets can influence and inform each other.

Early detection and early intervention are effective for ASD. To date, more than 100 interventions for ASD have been developed, but there is a lack of interventions that target their core symptoms (Figure 4). The goal of ASD treatment is to improve the individual's functioning and well‐being. Intervention therapy is more effective in improving ASD‐related symptoms (e.g., effective use of language) than ASD characteristics. Early interventions based on mature behavior analysis can help ASD acquire specific skills to address problem behaviors. Here, we reviewed recent meta‐analyses, reviews, and consensus on intervention approaches, focusing on approaches that are evidence based and have positive outcomes in some respects (Table 3). In addition, there are many ASD interventions that overlap with each other in terms of operationalization, and there is a tendency for interventions to learn from and integrate with each other, and for each class of approaches to be divided into different “subcategories,” as well as some important or emerging approaches (Figure 4). We also made a review in this section.

**TABLE 3:** Recommended behavioral and educational interventions for autism spectrum disorder.

No.	Author	Title	Recommended interventions
1	Xu et al. (2017)^265^	Expert consensus on early identification, screening and early intervention of children with autism spectrum disorders	① ABA ② TEACCH ③ ESDM ④ PRT ⑤ PACT ⑥ RIT ⑦ JA
2	Howes et al. (2018)^266^	Autism spectrum disorder: consensus guidelines on assessment, treatment, and research from the British Association for Psychopharmacology	① Psychological approaches: social learning program, behavioral and life‐skills interventions, cognitive‐behavioral interventions, facilitated communication ② Pharmacological treatment: serotonergic agents, glutamatergic agents, GABAergic agents, dopamine receptor blockers ③ Non‐pharmacological approaches: social‐communication interventions, behavioral interventions, alternative interventions
3	Sandbank et al. (2020)^267^	Project aim: autism intervention meta‐analysis for studies of young children	① Behavioral approaches: EIBI, DTI, PECS, PBS, ABA ② Developmental approaches: DF, HM ③ NDBI: ESDM, EMT, PRT, JA, SP, EG, RG ④ TEACCH ⑤ Sensory‐based interventions ⑥ Animal‐assisted interventions: EAAT ⑦ Technology‐based interventions: CAI, TTDVD
4	Hyman et al. (2020)^268^	Identification, evaluation, and management of children with autism spectrum disorder	① ABA ② Developmental relationship‐focus intervention ③ NDBI ④ Parent‐mediated treatment or training ⑤ Educational interventions: LEAP, TEACCH ⑥ Other therapeutic interventions: SLI, MT, HM
5	Gosling et al. (2022)^269^	Efficacy of psychosocial interventions for autism spectrum disorder: an umbrella review	① PMI ② TECH ③ SSG ④ DEV ⑤ CBT ⑥ NDBI ⑦ TEACCH
6	Hirota et al. (2023)^28^	Autism spectrum disorder: a review	① Behavioral approaches: EIBI, DTI ② Developmental approaches: FM, PACT ③ NDBI: ESDM, PRT, JASPER, PI ④ TEACCH ⑤ CBT ⑥ GSSIs ⑦ Pharmacological interventions: aripiprazole, risperidone, methylphe‐nidate, atomoxetine, extended‐release guanfacine, melatonin, oxytocin

Abbreviations: ABA, applied behavior analysis; CAI, computer‐assisted instruction; CBT, cognitive behavioral therapy; DEV, developmental interventions; DF, DIR/Floortime; DTI, discrete tracking instruction; EAAT, equine‐assisted activities and therapy; EG, engagement; EIBI, early intensive behavioral intervention; EMT, enhanced milieu teaching; ESDM, Early Start Denver Model; FM, Floortime model; GSSI, group social skills intervention; HM, Hanen models; JA, joint attention; JASPER, Joint Attention, Symbolic Play, Engagement and Regulation;LEAP, learning experiences and alternative programs for preschoolers and their parents; MT, motor therapies; NDBI, naturalistic developmental behavioral intervention; PACT, preschool autism communication trial; PBS, positive behavioral supports; PECS, picture exchange communication system; PI, parent involvement; PMI, parent‐mediated interventions; PRT, pivotal response treatment; RG, regulation; RIT, reciprocal imitation training; SLI, speech and language interventions; SP, symbolic play; SSG, social skill groups; TEACCH, treatment and education of autistic and related communication children; TECH, technology‐mediated interventions; TTDVD, the transportersTM DVD series.

### 4.5 Advances in behavioral and educational interventions

A recent review summarized evidence‐supported intervention approaches, including behavioral approaches (e.g., early intensive behavioral intervention [EIBI], discrete trial training), developmental approaches (e.g., developmental, individual differences, relationship‐based/Floortime model, preschool autism communication trial [PACT]), naturalistic developmental behavioral intervention (NDBI) (e.g., Early Start Denver Model [ESDM], pivotal response treatment [PRT], JASPER, Project ImPACT), treatment and education of autistic and related communication children (TEACCH), psychotherapy (cognitive behavioral therapy [CBT]), and group social skills interventions.^28^ In this review, the authors highlight NDBI, parent‐mediated interventions, CBT, and the fact that school‐aged children with ASD can often receive behavioral, speech, integration, and physical therapy in the school setting.^28^

A recent umbrella review identified several psychosocial interventions that are expected to improve symptoms associated with ASD at different stages of life, such as early reinforcement behavioral interventions, developmental interventions, natural developmental behavioral interventions, and parent‐mediated interventions that improve social communication deficits, overall cognitive abilities, and adaptive behaviors in children with ASD in preschool‐age children. The effectiveness of social skills groups in improving social communication deficits and overall ASD symptoms in school‐aged children and adolescents is supported by suggestive evidence.^269^ Another umbrella review identified positive therapeutic effects of behavioral interventions, developmental interventions, NDBI, technology‐based interventions, and CBT for several child and family outcomes.^270^

Moreover, a recent systematic review and meta‐analysis summarized the effects of seven early intervention types (behavioral, developmental, NDBI, TEACCH, sensory based, animal assisted, and technology based, aged between 0 and 8 years).^267^ Of these, significant positive effects were found for behavioral, developmental, and NDBI intervention types. When effect size estimates were limited to studies with a randomized controlled trial (RCT) design, there was evidence of positive summary effects for developmental and NDBI intervention types only. When effect estimates were limited to RCT designs and outcomes without detectable risk of bias, no intervention type showed a significant effect on any outcome.^267^ Together, despite the availability of multiple intervention models for children with ASD, many have still failed to demonstrate effectiveness in clinical trials. More well‐designed RCTs are still needed to gain a clearer understanding of the efficacy of these interventions^269^, ^271^ (Table 4).

**TABLE 4:** Related clinical trials related to interventions for autism spectrum disorders (ASD).

No.	Author	Study type	Clinical trial number	Sample size	Conclusion
1	Gabriels et al. (2015)^272^	Retrospective case	NCT 02301195	116	The study further establishes the evidence base supporting EAAT as a viable therapeutic option for children and adolescents with ASD. Further research is needed to examine the joint attention and movement experiences are key THR mechanisms to observe behavioral and social communication improvements in the ASD population.
2	Bearss et al. (2015)^273^	Retrospective case	NCT 01233414	30	Significant improvement (>12 units) in two patients and minor improvement (8–12 units) in eight patients.
3	Bieleninik et al. (2017)^274^	Retrospective case	ISRCTN 78923965	167	CBT was efficacious for children with ASD and interfering anxiety, an adapted CBT approach showed additional advantages. CBT can be considered as a professional reference for psychological treatment of autistic children.
4	Sharda et al. (2018)^275^	Retrospective case	ISRCTN 26821793	51	The study provides the first evidence that 8−12 weeks of individual music intervention can indeed improve social communication and function brain connectivity.
5	Grimaldi et al. (2018)^276^	Retrospective case	NCT 02720900	61	After 1 week of medication, all patients had significant improvements in abnormal behavior and irritability scores, with the risperidone group showing significant improvement at each assessment period.
6	DeVane et al. (2019)^277^	Retrospective case	NCT 01333072	364	ASD children who underwent improvisational music therapy and enhanced standard care showed improvement in scale assessment results, but compared with the two methods there was no significant difference in symptom severity based on the ADOS social affect domain over 5 months, indicating that the effect of using improvisational music therapy to reduce symptoms in ASD children was not significant.
7	Voss et al. (2019)^278^	Retrospective case	NCT 03569176	71	In terms of socialization, children who received the wearable intervention improved significantly than those who received only standard‐of‐care behavioral treatments, indicating potential for digital home therapy.
8	Malow et al. (2020)^279^	Retrospective case	NCT 01906866	80	Nightly pediatric prolonged‐release melatonin at optimal dose of 2, 5, or 10 mg is safe and effective for long‐term treatment in children and adolescents with ASD and insomnia, which has no detrimental effects on children's growth and pubertal development.
8	Wood et al. (2020)^280^	Retrospective case	NCT 02028247	150	A whole‐plant extract BOL‐DP‐O‐01‐W which contains CBD and THC in a 20:1 ratio improved disruptive behaviors on one primary outcome measures and on a secondary outcome, an index of ASD core symptoms, with acceptable adverse events.
9	Sikich et al. (2021)^281^	Retrospective case	NCT 01944046	277	In this trial involving children and adolescents with ASD, 24 weeks of daily intranasal oxytocin treatment, as compared with placebo, did not improve social interaction or other measures of social function related to ASD.
10	Aran et al. (2021)^282^	Retrospective case	NCT 02956226	30	Children on exclusion diets were less likely to report gastrointestinal abnormalities and had lower abundance of the Bifidobacterium and Veillonellaceae families but higher presence of Faecalibacterium and Bacteroidetes. A combined dietary approach resulted in significant changes in gut microbiota composition and metabolism.
11	Scahill et al. (2022)^283^	Retrospective case	NCT 02483910	83	On CELF, DI + TAU did not meet the prespecified difference from TAU. When adjusted for IQ, DI + TAU was superior to TAU on CELF at end point. DI + TAU was superior to TAU on CGI‐I.
12	Chu et al. (2023)^284^	Retrospective case	ChiCTR 2100053165	78	Potentially positive effects of nonwearable digital therapy plus LSP on core symptoms associated with ASD were found in the study, which leading to a modest improvement in the function of sensory, motor and response inhibition, while reducing impulsivity and hyperactivity in preschoolers with both ASD and ADHD, and VR‐CBT was found to be an effective and feasible adjunctive digital tool.

Abbreviations: ADHD, attention deficit hyperactivity disorder; ADOS, autism diagnostic observation schedule; CBD, cannabidiol; CBT, cognitive behavioral therapy; CELF, clinical evaluation of language fundamentals; CGI‐I, clinical global impressions‐improvement scale; DI, direct instruction language for learning; EAAT, equine‐assisted activities and therapies; LSP, learning style profile; TAU, treatment as usual; THC, tetrahydrocannabinol; THR, therapeutic horseback riding; VR‐CBT, virtual reality‐incorporated cognitive behavioral therapy.

When developing a consensus, Chinese experts selected and recommended methods that are supported by randomized controlled studies, have a high level of evidence‐based medical evidence, and have a recommendation rating of “strongly recommended” for children with ASD under the age of 3 years and are eligible for implementation in China. The early intervention methods that are supported by randomized controlled studies have evidence‐based ratings and “strongly recommended” ratings for children with ASD under 3 years of age and are eligible for implementation in China, including ESDM, PRT, PACT, reciprocal imitation training, and joint attention (JA) training.^265^

Furthermore, it is also worth mentioning a recent report by the Lancet Commission, which states that individualized, stepped care strategies can meet an individual's needs throughout the life course, leading to effective assessment and care. The importance of community and family supports in lifelong intervention and treatment for individuals with autism. It further describes the broad spectrum of autism and introduces the concept of “profound autism”; that is, “profound autism” should be paid attention to.^285^

### 4.6 Methods of behavioral intervention

#### 4.6.1 Applied behavior analysis

Over the past decades, applied behavior analysis (ABA) has been at the forefront of these interventions and has been recommended as a scientifically validated intervention in different countries.^286^ Due to its high level of acceptance, ABA interventions have also become the benchmark for existing and subsequently developed interventions. In most studies, this approach has shown positive improvements in cognition, language development, social skills and communication, and adaptive behavior in children with ASD, along with reductions in problem behaviors.^287^

EIBI was the first intensive ABA therapy proposed for ASD, focusing on eliminating atypical behaviors and building learning capacity. Since then, treatments for ASD have weakened structural features while focusing on more complex cognitive and social skills.^288^, ^289^ The EIBI model relies heavily on discrete tracking instruction (DTI), which focuses on reducing extraneous details and teaching skills and learning content in a repetitive and streamlined manner. Ongoing data collection and analysis are key components of DTI,^290^, ^291^ and these data are an important reference for determining how quickly children progress and whether program modifications are needed. In general, DTI is more appropriate for developing JA, play, or imitation skills in children around 2 years of age,^292^ and may also be of shorter duration as conditions improve to address more complex social behaviors.^293^

One of the earliest alternative forms of ABA for ASD was the Natural Language Paradigm (NLP), the earliest natural language training strategy,^294^ whose main advantage was the integration of therapy into natural, ongoing social, and play activities. PRT^295^ and ESDM are the naturalistic language strategies with the most empirical evidence to support their effectiveness. As an extension of NLP, the training goals of PRT focus on motivation to interact with others, self‐management, self‐regulation, and response to multiple cues.^296^ Its validity has been supported by several studies.

#### 4.6.2 Physical exercise

Studies have found that children with ASD spend significantly less time per day participating in moderate to vigorous physical activity compared to normally growing children.^297^ Physical activity of appropriate intensity is a remedy to reduce physical–motor deficits, stereotypic and aggressive behaviors, and improve cognitive functioning in individuals with ASD.^298^, ^299^, ^300^ In recent years, there has been an explosion of systematic evaluations and meta‐analyses of exercise interventions on stereotypic behaviors, executive functions, and cognitive abilities in children and adolescents with ASD.^298^, ^299^, ^301^, ^302^, ^303^ Stereotypical behavior patterns of individuals with ASD are alleviated through exercise intervention.^304^, ^305^ It is also beneficial to enhance overall cognitive flexibility and inhibitory control,^301^ and reduce the deficits of social interaction.^299^ Different types of exercise all play a positive role in alleviating stereotypical behaviors in people with ASD.^306^, ^307^, ^308^, ^309^, ^310^, ^311^, ^312^, ^313^, ^314^, ^315^, ^316^, ^317^, ^318^ Although the molecular mechanisms involved in the beneficial effects of exercise on ASD remission are still unknown, the thesis that cytokines released after exercise play an important role in regulating neuronal metabolism, neuroinflammation, and neuroplasticity has been confirmed,^316^, ^319^, ^320^ which may be related to the improvement of symptoms in children with ASD and associated comorbidities.^316^

## 5 METHODS OF EDUCATIONAL INTERVENTION

### 5.1 Music therapy intervention

There is a long history of using music or music therapy services for non‐musical goals (including social skills) for people with ASD.^321^ Currently, most music therapy applications for ASD are focused on children and adolescents; they are thought to have positive effects on social skills, including engagement behaviors,^322^ increased emotional involvement,^323^ improved social interactions,^324^, ^325^ increased social greeting routines,^326^ JA behaviors,^327^, ^328^ peer interactions,^329^ communication skills,^274^, ^330^, ^331^ and cognitive social skills.^332^

There are also differences in the effectiveness of different types of music therapy for people with ASD. Improvisational music therapy (IMT) is one of the most studied music therapies for children with ASD.^327^, ^333^, ^334^, ^335^, ^336^ Family‐centered music therapy as an important variant of IMT improves social interactions in families, communities, and parent–child relationships.^337^ However, studies have also reported contradictory results or no improvement in some areas.^338^, ^339^ Nevertheless, the feasibility of music therapy interventions for children with ASD has received preliminary support, at least in terms of improving social interaction, verbal communication, initiating behavior, and social–emotional reciprocity.

### 5.2 Play therapy intervention

Providing children with ASD the opportunity to engage in play activities can strengthen their connections with others and improve social interaction deficits. Patient‐centered play therapy is considered an effective evidence‐based intervention to improve core issues related to ASD, such as social skills, communication, emotion regulation, and JA,^340^, ^341^, ^342^, ^343^ while a reduction in repetitive behaviors is a strong reason for the validation of the effectiveness of play therapy.^344^

### 5.3 Family involved intervention

Research has shown that involving parents in interventions reinforces the effectiveness of the intervention and the prevalence of skills outside of the school setting.^345^ Family–school partnerships (FSPs) are a child‐centered approach, where families and school collaborate and coordinate to produce positive student outcomes in the social, emotional, behavioral, and academic domains.^346^, ^347^ Active parental involvement in education and intervention can have a significant impact on children's learning and development, children's cognitive and language skills,^348^ school participation, academic achievement,^349^ and children's problem‐solving skills can be improved and enhanced.^350^ In addition, parental involvement can lead to positive outcomes in prosocial behavior,^351^ peer interaction, and self‐regulatory skills.^352^ In addition to the FSPs mentioned above, parent involvement (PI) is also applicable to family‐level interventions and education for children with ASD. Unlike FSP, PI focuses more on the structure and process of activities.^353^, ^354^ Numerous studies have shown that in children with ASD, improvements in social communication and reductions in restrictive and repetitive behaviors occur after interventions using the PI model.^355^, ^356^, ^357^

### 5.4 The interventions derived from technical devices for ASD

With the rapid development of modern technology, a number of assistive devices for the rehabilitation of people with autism have been developed and put into use. These devices have shown some effectiveness in ASD interventions and deserve further study and evaluation.

#### 5.4.1 Speech‐generating device intervention application

Speech‐generating device (SGD) is a portable electronic device that displays various graphic symbols or written language and generates digital or synthetic speech.^356^, ^357^ For children with ASD, whose communication skills are severely lacking, the motor skills tolerance of SGD, the popularity of the output language, and the large storage space make it more socially acceptable.^358^, ^359^ At the same time, the SGD's ability to request, tag, comment, and answer questions extends its scope of application.^360^ Previous studies have shown that SGD can improve participants' communication skills,^358^, ^361^, ^362^ while the acquisition of communication skills is a top priority in early intervention programs for ASD.

#### 5.4.2 Virtual reality technology application

Virtual reality (VR) is a realistic and immersive three‐dimensional virtual environment created by interactive software and hardware and is a product of multidisciplinary integration. With the increasing sophistication of VR technology, researchers have successfully applied it to the treatment of people with autism.^363^, ^364^, ^365^, ^366^, ^367^, ^368^ On this basis, immersive virtual reality has been developed, which is able to reproduce real objects and scenes to a higher degree.^369^, ^370^, ^371^, ^372^ However, VR still has some shortcomings, such as the current VR technologies used in ASD treatment are homogeneous and usually can target only one characteristic, and VR simulation scenes are still different from reality. It is expected that VR technology will continue to overcome its limitations and meet the individual needs of people with autism.

#### 5.4.3 Social bots’ application

In contrast to VR, another more tangible technological development, humanoid robot, is also being used for the treatment of ASD. There is growing evidence that robotic assistance has a positive effect on the improvement of the condition of individuals with ASD.^373^, ^374^, ^375^, ^376^, ^377^ Unlike humans, robots operating in predictable and legitimate systems provide a highly structured learning environment for people with ASD, enabling structured and standardized interventions that will help them focus on relevant stimuli, and certain social behaviors may be simulated in the standardized social contexts created by such structured interactions.^378^, ^379^ In the field of autism, there are precedents for the use of robots to assist in the diagnostic process, improve eye contact and spontaneous interaction, turn‐taking activities, mimicry, emotion recognition, JA, triadic interactions, etc.^373^, ^376^, ^380^ The results of an induction training for people with ASD involving android robots are also encouraging and make other approaches to intervention using robots worth trying.^381^

#### 5.4.4 Medical intervention and potential drug target

Currently, there is still a lack of drugs to treat the core symptoms of ASD, and research on them is difficult.^382^, ^383^ Here, we provide an overview of existing pharmacologic therapies for ASD as well as those that target its common pathophysiology and gut microbiota. With the rapid growth of genomics and systems neuroscience, a variety of new molecular targets are surfacing.^384^

#### 5.4.5 Drug treatment for ASD

There are currently no medications available worldwide that specifically target the core symptoms of ASD. More commonly, existing antipsychotics are used to alleviate anxiety,^385^ depression,^386^, ^387^ or obsessive–compulsive disorder^388^ in order to ameliorate certain symptoms of ASD, such as ADHD.^389^, ^390^ The US Food and Drug Administration (FDA) has approved two medications, risperidone and aripiprazole, for the pharmacologic treatment of ASD‐related irritability and aggression.^391^ However, while there are medications that can alleviate several specific conditions of ASD, the side effects should not be underestimated. For example, aripiprazole can cause side effects, such as drowsiness/sedation, increased sleep duration, and weight gain.^392^ In addition, selective 5‐hydroxytryptamine reuptake inhibitors have been approved by the FDA for a wide range of other disorders, and as a result they are frequently and increasingly used in the treatment of ASD.^393^ A recent review based on RCTs suggests that the following medications improve at least one core symptom area compared to placebo: aripiprazole, atoxetine, bumetanide, and risperidone for children/adolescents, and fluoxetine, fluvoxamine, oxytocin, and risperidone for adults.^394^, ^395^

Consequently, finding common mechanisms to screen for access to targeted drugs remains important and possible.^396^, ^397^ For example, and clinical trials are attempting to use the GABAergic system as a therapeutic strategy for ASD,^396^ and a recent study showed that a clinically relevant selective ERK pathway inhibitor reverses the core deficits in a mouse model of autism.^397^

### 5.5 Interventions and treatments related to common pathological mechanisms

Given that inflammation and immunity, oxidative stress, and mitochondrial dysfunction are common pathophysiological mechanisms of ASD, there are a number of proposals and studies targeting them, including antioxidant, anti‐inflammatory, immunomodulatory, and improving mitochondrial function and metabolism.^398^, ^399^, ^400^

Several clinical studies on antioxidant therapy for ASD have been reported, including radicicicol,^401^ resveratrol,^402^ coenzyme Q10,^403^ N‐acetylcysteine (NAC),^404^ omega‐3 fatty acids,^405^ arachidonic acid, and docosahexaenoic acid (DHA),^406^ all of which showed beneficial effects except for resveratrol, whose role is uncertain. Of these, NAC appears to be the most effective antioxidant therapy.^400^ In addition, some studies have demonstrated that supplementation with micronutrients related to redox metabolism (e.g., methyl B12) can be helpful for children with autism.^407^ Other studies have evaluated antioxidant‐rich foods, including broccoli,^408^ camel's milk,^409^ and dark chocolate.^410^ Notably, there are antioxidants, such as radicicchioidin, resveratrol, naringenin, curcumin, and guanidinium that are not only antioxidants, but also activators of Nrf2, a transcription factor involved in immune dysregulation, inflammation, oxidative stress, and mitochondrial dysfunction.^411^

However, many of the oxidative stress treatment groups in the study showed strong individual differences, reflecting the heterogeneity of ASD.^412^ Therefore, assessing and identifying physiological changes associated with ASD and taking targeted and personalized interventions are more likely to produce positive treatment outcomes.^398^, ^412^ As mentioned in a recent review,^398^ folic acid supplementation has a positive effect in individuals with ASD identified by autoantibodies to the folate receptor,^413^ whereas methylcobalamin has significant clinical utility when impaired methylation capacity.^414^, ^415^ Mitochondrial regulatory cofactors should be considered when mitochondrial dysfunction is evident. Multivitamin/multimineral formulas, as well as biotin, appear to be appropriate when metabolic abnormalities have been identified, as well as the use of low‐dose suramin antipurinergic therapy.^416^

In addition, many antioxidant molecules available in nature show anti‐inflammatory activity.^417^ Some natural antioxidants have been carried out in human studies, such as GSH, vitamin C, NAC, flavonoids, luteolin, quercetin, rutin,^418^, ^419^ palmitoylethanolamide and luteolin,^420^ DHA, eicosapentaenoic acid (EPA),^421^ and Ginkgo biloba extract 761.^422^ The most common of the inflammatory signaling pathways is nuclear factor‐κB (NF‐κB), MAPK, and JAK–STAT pathways.^423^ Several preclinical studies have been initiated targeting these pathways, including resveratrol,^424^ palmitoylethanolamide, and luteolin^420^ against the NF‐κB pathway, and luteolin, diosmine,^425^ and quercetine^426^ against the janus kinase/signal transducer and activator of transcription (JAK/STAT) pathway, as well as IL‐17A antibody against ERK/MAPK pathway.^427^, ^428^

Marchezan et al. classified immune and inflammatory interventions for ASD into two broad categories: (1) using radicicicol, celecoxib, lenalidomide, hexacosanolide, spironolactone, flavonoid lignocerotonin, corticosteroids, oral immunoglobulins, intravenous immunoglobulins, and cellular therapy. (2) Other ASD therapies that have been used or are being studied that are initially characterized as neither anti‐inflammatory nor immunomodulatory at first, but exhibit immunomodulatory capabilities throughout the course of treatment: risperidone, vitamin D, omega‐3, ginkgo biloba, l‐creatinine, n‐acetylcysteine, and microbiome recovery.^429^ Another narrative review of randomized controlled placebo trials summarizes how immunomodulatory/anti‐inflammatory therapeutic agents such as prednisolone, pregnenolone, celecoxib, minocycline, n‐acetylcysteine, radicicic acid, and/or omega‐3 fatty acids may be useful in the core management of (e.g., stereotypic behaviors) and related (e.g., irritability, hyperactivity, lethargy) symptoms in individuals with autism.^430^ Likewise, a review based on RCTs concluded that myostatin, haloperidol, folinic acid, guanfacine, omega‐3 fatty acids, probiotics, radicicic acid, sodium alginate, and sodium valproate showed some signs of improvement, but were imprecise and unreliable.^431^

Overall, among the several intervention approaches described above, attention needs to be paid to individualization, targeting interventions to subgroups of ASDs with associations with these pathophysiological mechanisms, and improving the efficacy of interventions.^412^, ^432^ The literature on intervention efficacy is limited, and large‐scale RCTs are still needed to provide strong evidence as well as the use of biomarkers.^430^

#### 5.5.1 Interventions for ASD targeting the microbiota‐gut‐brain axis

As research into the mechanisms associated with gut microbial imbalance and the development of ASD has intensified, probiotics, prebiotics, fecal microbiota transplantation (FMT), microbiota transfer therapy, antibiotics, and diet dietary adjustment methods received considerable attention.^433^ The beneficial effects of probiotics in improving mood and regulating host behavior have been explored, with specific probiotic therapy reducing the severity of ASD symptoms and developing strategies to manage typical social impairment, communication disorders, perceptual impairment, and behavioral limitations.^434^, ^435^, ^436^, ^437^, ^438^, ^439^ FMT has been shown to be an established and effective treatment for recurrent *Clostridium difficile* infection.^440^ It has also been proposed as a safe and effective strategy to modulate the symbiosis of the gastrointestinal microbiota and improve behavioral symptoms in children with ASD.^440^, ^441^ Recently, a study showed that FMT improved VPA‐induced ASD mice by modulating 5‐hydroxytryptaminergic and glutamatergic synaptic signaling pathways.^442^ Modified FMT therapy for children with ASD resulted in significant improvements in gastrointestinal symptoms and ASD symptoms, and follow‐up of these individuals after 2 years showed that most of the improvements in gastrointestinal symptoms were maintained, including significant increases in bacterial diversity and relative abundance of beneficial bacteria such as bifidobacterial.^440^, ^443^ However, due to the complexity of the intestinal microbiota, FMT therapies are still highly heterogeneous with respect to donor selection, material preparation, ideal dosing regimen, and cost‐effectiveness, and are still a long way from clinical application. In addition, although these therapeutic modalities have been shown to be safe and effective for short‐term supplementation, the safety over long periods of time remains uncertain needs to be validated by additional studies.^195^ Overall, autism interventions targeting the gut–brain axis have the potential to be an effective treatment for ASD and are expected to have a positive effect on the improvement of ASD symptoms.^444^

## 6 CONCLUSION AND PERSPECTIVES

ASDs have become a common neurological developmental disorder in children. Early detection and early intervention are highly effective. Heterogeneity is a distinctive feature of children with ASD. In addition to the core symptoms, children with ASD are accompanied by different behavioral abnormalities and comorbidities with varying degrees of severity, which exacerbates its complexity and poses challenges for its research and clinical translation.

In this paper, based on the review of the pathological mechanisms of ASD, the progress of its diagnostic markers and intervention methods are reviewed. ASD is caused by genetic and environmental factors and their interactions, its signaling pathways, and mechanisms are convergent. Oxidative stress, inflammation and immunity, mitochondrial dysfunction, and intestinal flora dysregulation are common pathophysiological mechanisms, and they are interrelated. There are also common mechanisms between ASD and comorbidities. These provide the basis for the diagnosis and treatment, at least on a stratified or subclass‐based basis. Stratified biomarkers are objective measures used to define subgroups of individuals with common biological characteristics. The treatment and management of children with ASD often involves the management of associated medical problems and psychopathological comorbidities. Therefore, it is important to consider both commonalities and individuality in diagnosis, treatment, and intervention of ASD. Priority is given to personalized diagnosis and treatment for different individuals to improve the precision and efficacy of ASD diagnosis, treatment, and rehabilitation. With the application of high‐throughput omics, such as genomics, proteomics, metabolomics, transcriptomics, as well as in‐depth mechanism studies, it is expected to find common mechanisms among individuals with ASD subjects and find specific early diagnostic biomarkers and drug therapeutic targets, which is a key research direction in the future. In terms of ASD intervention and treatment, large‐scale RCT‐based clinical studies need to be strengthened. In this context, maximizing the sample pools, designing studies with more diverse populations, increasing the number of subjects in RCTs, and defining more accurate patient codifies using gold standard diagnostic instruments are common themes for future developments in the field.

In summary, ASD is a highly heterogeneous, and it is particularly important to improve the understanding of the biological basis of the inherent heterogeneity of ASD, to search for potential common or convergent mechanisms, and to explore the “homogeneity” within the “heterogeneity.” On this basis, the development of diagnosis, intervention and treatment is an effective way to achieve accurate diagnosis and treatment of ASD.

## AUTHOR CONTRIBUTIONS

All authors were involved in the conceptualization and design of this paper. Preparation of relevant materials, data collection, and analysis were also performed by all authors. The first draft of the manuscript was written by H.Z., Z.L., G.M., and A.Q. L.S., X.R., X.L., X.Y., and C.F. undertook the revision of the manuscript. All authors read and approved the final manuscript.

## CONFLICT OF INTEREST STATEMENT

All authors read and approved the final manuscript and declare they have no conflicts of interest.

## ETHICS STATEMENT

Not applicable.

## Supporting information

Supporting Information

## Data Availability

Data availability is not applicable to this review as no new data were created or analyzed in this study.
