# Supplementary material for: Autism spectrum disorder: pathogenesis, biomarker, and intervention therapy
Source: MedComm (2020). 2024 Mar 2;5(3):e497. doi: 10.1002/mco2.497 (PMC10908366; doi:10.1002/mco2.497)
Supplement: Supplementary file 1 — Supporting Information [file MCO2-5-e497-s001.pdf]

# Supplementary Data

## Autism spectrum disorder: pathogenesis, biomarker and intervention therapy

Hongbin Zhuang[1]#, Zhiyuan Liang[1]#, Guanwei Ma[1], Ayesha Qureshi[1], Xiaoqian Ran[1], Chengyun Feng[2], Xukun Liu[1], Xi Yan[1], Liming Shen[*,1,3]

1 College of Life Science and Oceanography, Shenzhen University, Shenzhen 518071, P. R. China

2 Maternal and Child Health Hospital of Baoan, Shenzhen 518100, P. R., China

3 Shenzhen-Hong Kong Institute of Brain Science-Shenzhen Fundamental Research Institutions, Shenzhen 518055, P. R. China

*Corresponding Author: Liming Shen

College of Life Sciences and Oceanography, Shenzhen University, Shenzhen 518060, P. R. China

E-mail: slm@szu.edu.cn

Phone: +86-755-26907449

Fax: +86-755-26534274

# Hongbin Zhuang and Zhiyuan Liang have contributed equally to this study.

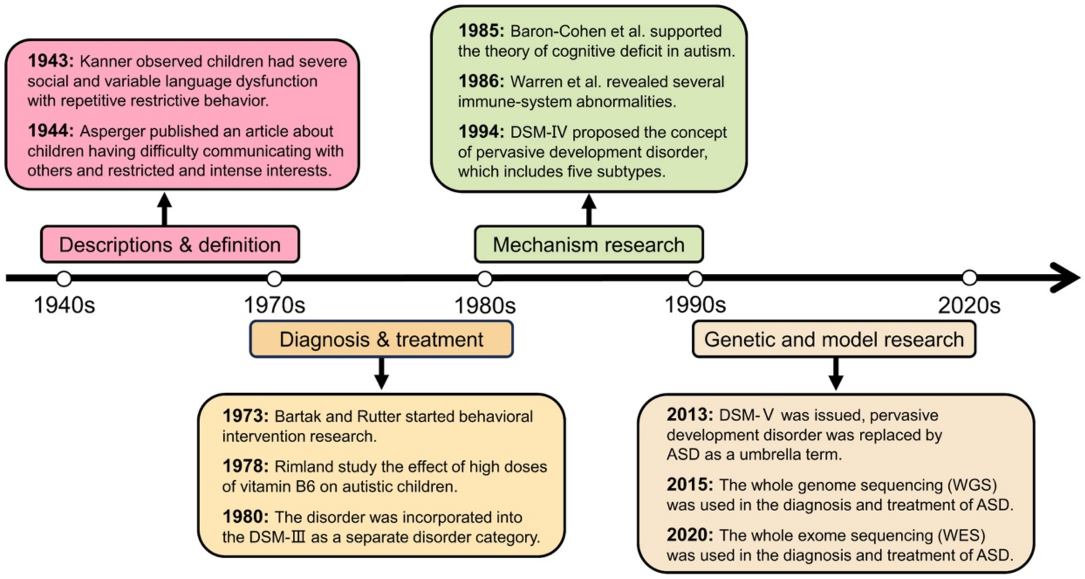


**Figure S1. The milestone events associated with autism spectrum disorder.** Original description of autism was in 1940s, subsequently leading to a series of studies on the definition, diagnosis and treatment of autism in 1960s and 1970s. From the behavioral intervention research in 1973, people began to explore ways to effectively intervene in the disorder. Up to now, advances in whole genome sequencing (WGS) and whole exome sequencing (WES) have revealed patterns of inheritance and the types of genetic variation that result in ASD and studies in models have identified a mountain of evidence for molecular mechanisms for ASD.

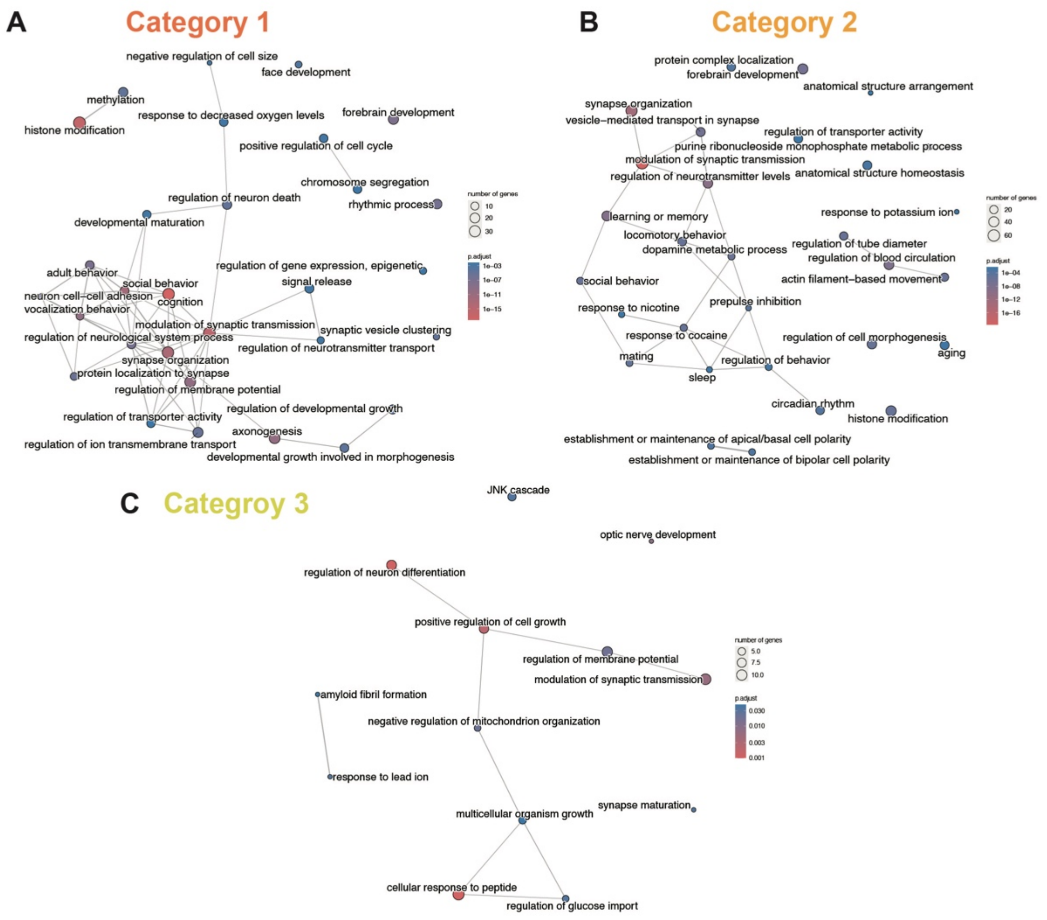


**Figure S2. The network analysis of ASD risk genes from SFARI database.**

The risk genes ASD are classified into three grades based on SFARI database and analyzed respectively. The significant pathways are involved in interaction network. The size of the circle represented the number of risk genes contained in the pathway and the line between circle indicated that two pathways have an interaction based on Gene Ontology (GO) database. The pathway which placed in core location showed a wide interaction. The enriched analysis was performed on R packages Clusterprofiler (v4.2.1).

**Table S1. Convergent pathways related to ASD pathology by omics methods.**

| No. | Author | Method | Converged pathway | Sample | Gene type | Major finding |
|---|---|---|---|---|---|---|
| 1 | Niere, F., et al. | Proteome | PI3K/Akt/mTOR | Rat brain | - | 1. Both ASD and epilepsy phenotypes and has overactive mTORC1 signaling.<br>2. A brief repression of mTORC1 activity leads to a significant remodeling of synaptic proteins. |
| 2 | Lombardo, M.V., et al. | Transcriptome | PI3K/Akt/mTOR | Mouse brain | - | 1. MIA induced transcriptional dysregulation of mTOR and EIF4E-dependent signaling. |
| 3 | Mencer, S., et al. | SNO-proteome | PI3K/Akt/mTOR | Mouse brain | InsG3680(+/+) | 1. The mammalian target of mTORC1 signaling pathway as one of the shared molecular mechanisms. |
| 4 | Wesseling, H., et al. | Proteome | PI3K/Akt/mTOR | Mouse brain | Tsc1+/− | 1. proteomic alterations in the hippocampus validated the pathways “myelination”, “dendrite,” and “oxidative stress”, an upregulation of ribosomal proteins and the mTOR kinase. |
| 5 | Wan, H., et al. | Proteome | PI3K/Akt/mTOR | Mouse brain | *wdr45* KO | 1. Proteomics anlyais reveals the accumulation of ER proteins in *wdr*45 KO mice<br>2. Suppression of ER stress or activation of autophagy through MTOR inhibition alleviated cell death. |
| 6 | Gazestani, V. H., et al. | Transcriptome | PI3K/Akt/mTOR | Human leukocyte | - | 1. Whie the majority of rASD genes are not fully penetrant to ASD, the regulatory consequence of many rASD genes on the DE-ASD network are channeled through the PI3K/AKT, RAS/ERK, WNT/β-catenin signaling pathways. |
| 7 | Matic, K., et al. | Phosphoproteome | ERK/MAPK signal | Mouse brain | Fmr1 KO | 1. Downregulation of the MEK/ERK pathway are obeserved in the absence of FMRP, with decreased phosphorylation on ERK1/2.<br>2. Several key proteins from the p53 pathway were detected, pointing to the involvement of p53 signaling in dysregulated cell cycle control in Fragile X syndrome. |
| 8 | Yang, J., et al. | Proteome | ERK/MAPK signal | Human plasma | - | 1. The study showed increased levels of MAPKAPK3 and MRPL33 in ASD.<br>2. MAPK/ERK signaling pathway and mitochondrial dysfunction are involved in the pathogenesis of ASD |
| 9 | Berg, J. M., et al. | Proteomic interactome | JAKMIP1 pathway | Mouse brain | - | 1. JAKMIP1 interacted with proteins related to signaling and interaction, nervous system development and function, and protein synthesis. |
| 10 | Nishimura, Y., et al. | Transcriptome | JAKMIP1 pathway | Human lymphoblastoid cells | - | 1. This study identified common regulation of two other dysregulated genes, JAKMIP1 and GPR155, downstream of FMR1 or CYFIP1 |
| 11 | Reilly, J., et al. | Proteome / Transcriptome | Calcium signaling | Human, mouse and rat datasets | - | 1. Calcium signalling and the glutamatergic synapse were found to be highly interconnected among pathways in the combined geneset.<br>2. Converging ASD candidate genes on E/I balance and calcium signalling pathway. |
| 12 | Wen, Y., et al. | Network anlaysis | ERK/MAPK signal / Calcium signaling | Human databases | - | 1. Calcium signaling pathways and MAPK signaling pathway are interactive hubs with other pathways.<br>2.The analysis showed convergent indications that the process "calcium-PRC (protein kinase C)-Ras-Raf-MAPK/ERK" is likely a major contributor to ASD pathophysiology. |
| 13 | Baucum, A. J., et al. | Proteome / Interactome | Calcium signaling | Mouse brain | - | 1. Significantly more CaMKAPs co-precipitated with WT CaMKII holoenzymes in the synaptic fraction compared to the membrane fraction, with functions including scaffolding, microtubule organization, actin organization, ribosomal function, vesicle trafficking, and others. |

Abbreviation: ASD, autism spectrum disorder; E/I, excitation/inhibition; ER, endoplasmic reticulum; KO: kncok out; MIA, maternal immune activation; SNO, S-nitrosylation; WT, wild type.

**Table S2. Non-targeted metabolomics study of intestinal microbial metabolites as biomarkers of ASD**

| No. | Author | Sample | Method | Related Metabolites<br>(#：ASD compared to Ctrl,<br>*：ASD compared to TD) | Metabolic Process Involved |
|---|---|---|---|---|---|
| 1 | Wang et al. (2012)[222] | Fecal | HPLC-GC | **Increased#:** Acetic, Butyric, Isobutyric, Propionic acid, Valeric, Isovaleric, Total chain fatty acid, SCFA. | Intestinal permeability, Total SCFA metabolism, Gut microbiota metabolism. |
| 2 | Angelis et al. (2013)[215] | Fecal | GC-MS/ SPME | **Decreased#:** GABA, Ethanol, SCFA1-Pentanol2-propyl-1-pentanol.<br>**Increased#:** Pentanoic, 3,7-Dimethyl-2,6-Octadien-1-ol, P-cresol, Acetic acid methyleseter, Indole, 3Methylindole, Acetic, Propionic acid. | Metabolism of gut microbiota,<br>FAA metabolism |
| 3 | Kang et al. (2018)[223] | Fecal | 1NMR spectroscopy | ASD compared to Neurotypical:<br>**Decreased:** Lactate, Formate, Acetate.<br>**Increased:** P-cresol, Caprate, Isopropanol. | Respiratory depression, the homeostasis of colonic epithelial cells |
| 4 | Wang et al. (2019)[224] | Fecal | LC-MS/MS | **Decreased*:** 2-Keto-glutaramic acid, Palmitic amide, Glutamate, Fumaric acid, Epinephrine, Cinnamicacid, Benzaladehyde, Cortisol, L-Aspartic acid<br>**Increased*:** Chenodeoxycholic acid 3-sulfate, Taurocholic acid. | Composition of gut microbiota,<br>Intestinal hormone metabolism |
| 5 | Wang et al. (2020)[225] | Fecal | GC-MS; UHPLC-MS /MS | **Decreased#:** Acetic acid, Propionic acid, Butyric acid, Homovanillic acid.<br>**Increased#:** Serotonin, SCFAs. | Ecological imbalance of gut microbiota, Disorder of dopamine metabolism |
| 6 | Dan et al. (2020)[226] | Fecal | LC/MS | **Decreased*:** Tetracosahexaenoic acid, 2'-lndoleacetaldehyde, Acetaldehyde, Deoxyguanosine, Adenine, Xanthine, lmidazo4e-4-acetaidhyde.<br>**Increased*:** Desaminotyrosine, Chloroneb, Hexanoic acid, Cytosine, Deoxyinosine, Glutamylproline, DL-2 aminooctanoic-acid, 2,5-Dioxopentanoate, 2'Deoxyuridine. | Phenylalanine and Tyrosine metabolism, Arginine and proline metabolism, Histidine and Aspartate metabolism, Glutamate metabolism |
| 7 | Xiao et al. (2021)[183] | Fecal | LC-MS/MS | **Decreased*:** 6-Hydroxymelatonin, 5-Hydroxyindole-3-acetic acid<br>**Increased*:** Tryptamine, 5-Hydroxytryptophan, Serotonin, 5-Hydroxy-N-formylkynurenine, | Intestinal microbiota, Tryptophan metabolism, Serotonin metabolism. |
| 8 | Zhu et al. (2022)[187] | Fecal | LC-MS/MS | **Decreased*:** TPP, DHF, 5MTHF, Retinol, Vitamin C, L-Ascorbic acid, Dihydrofolic acid, Lumichrome, Pyridoxamine.<br>**Increased*:** All-trans-retinal, B,e-carotene-3,3-diol, Tocopherol, 5-Hydroxytryptophan, Serotonin, Homocysteine, Xanthurenic acid, 4'-apo-beta- | Tryptophan metabolism, Retinol metabolism, Vitamin digestion and absorption. |

| | | | | | |
|---|---|---|---|---|---|
| | | | | carotenal. | |
| 9 | Kuwabara et al. (2013)[227] | Blood | CE-TOF-MS | **Decreased#:** 5-Oxoproline, Lactic acid.<br>**Increased#:** Taurine, Arginine. | Oxidative stress, Mitochondrial disorder |
| 10 | West et al. (2014)[216] | Blood | GC-MS, LC-HRMS | **Decreased*:** Citric acid, Fatty acids, Creatinine, the branched chain amino acid isoleucine, Homocitrulline.<br>**Increased*:** Succinic acid, 3-Aminoisobutyric acid, Aspartic acid, Glutamate, DHEAS, Glutaric acid. | Mitochondrial disorder, Oxidative phosphorylation, Changes in gut microbiome, Transport and accumulation of metabolites in urine. |
| 11 | Wang et al. (2016)[228] | Blood | UPLC/Q-TOF MS/MS | **Decreased#:** Adrenic acid, L-acetylcarnitine, Acetylcarnitine, Uric acid, Arachidonic acid, Docosahexaenoic acid, Docosapentaenoic acid.<br>**Increased#:** LPA, LysoPE, Phytosphingosine, Pregnanetriol, 9,10-epoxyoctadecenoic acid, Sphingosine 1 phosphate. | Fatty acids β Oxidation, Mitochondrial dysfunction. |
| 12 | Anwar et al. (2016)[229] | Blood | HPLC, LC-MS/MS | **Decreased#:** Thiamine Pyrophosphate. | Interorganizational, Exchange of metabolites, Mucosal immunity, Host microorganisms. |
| 13 | Jory et al. (2016)[230] | Blood | GLC | **Decreased#:** Docosahexaenoic acid, Arachidonic acid, Linoleic acid, Eicosapentaenoic acid, ω-3/ω-6 ratios. | Abnormal Fatty acid Metabolism, Cobalamin metabolism, Intestinal microbiota, Propionic acid production |
| 14 | Grayaa et al. (2018)[231] | Blood | GC–MS | **Decreased#:** 7α-Hydroxycholesterol, 25-Hydroxycholesterol.<br>**Increased:** 24-Hydroxycholesterol. | Cholesterol metabolism, Cytotoxicity, Oxidative stress, Cell apoptosis, Synaptic dysfunction |
| 15 | Rangel et al. (2019)[232] | Blood | UPLC–MS/MS | **Decreased#:** Glutamate, Nicotinamide.<br>**Increased#:** Isovalerylcarnitine, Isobutyrylcarnitine, Tryptophan, kynurenine, N-methyl-2-pyridone 5-carboxamide, 3-Indole sulfate, 1-methyl Nicotinamide, 5-Bromophosphatidylcholine Indolelactate. | Amino acid, Lipid, Nicotinamide metabolism, Oxidative stress |
| 16 | Wang et al. (2020)[225] | Blood | UHPLC-MS /MS | **Decreased#:** Homovanillic acid, Kynurenine.<br>**Increased#:** 5-HT, L-glutamine, L-arginine, L-histidine, 5-hy- droxyindoleacetic acid | Intestinalmicrobiota, Dopaminemetabolism, Tryptophan metabolism. |
| 17 | Kang et al. (2020)[219] | Blood | LC-MS | **Decreased*:** IMP, Iminodiacetate, Sarcosine, Methylsuccinate, Valylglycine, Leucylglycine, | Mucosal homeostasis, Energy production, |

| | | | | | |
|---|---|---|---|---|---|
| | | | | Galactonate, Nicotinamide riboside.<br>**Increased*:** Caprylate, Heptanoate, p-cresol sulfate | Folate metabolism. |
| 18 | Needham et al. (2021)[233] | Blood | LC-MS/MS | **Decreased*:** saturated fatty acid levels, long-chain acyl-carnitines.<br>**Increased*:** Cresol derivatives, 4EPS, 4-Methylbenzene sulfonate, 2-Ethylphenyl sulfate, 4-allylphenyl sulfate. | Oxidative stress, Mitochondrial dysfunction, Hormone level elevations, Lipid profile changes, Phenolic microbial metabolites. |
| 19 | Yap et al. (2010)[234] | Urine | 1H NMR Spectroscopy | **Decreased#:** Glutamate, Hippurate, PAG.<br>**Increased#:** Taurine, Acetate, DMA, NAG, Succinate, NMNA, NMND, 2PY. | Gastrointestinal Dysfunction, Oxidative stress, Energy metabolism, Inflammation, Sleep disorder. |
| 20 | Ming et al. (2012)[235] | Urine | UPLC/MS/MS | **Decreased#:** Glycine, Serine, Threonine, Alanine, Histidine, glutamyl amino acids, The organic acid, Taurine, Antioxidants, 3-(3-Hydroxyphenyl) propionate, 5-Aminovalerate.<br>**Increased#:** 2-(4-hydroxyphenyl) propiona, Taurocholenate sulfate. | Abnormal amino acid metabolism, Increased oxidative stress, Changes in gut microbiome. |
| 21 | Diémé et al. (2015)[236] | Urine | UPLC–HRMS, NMR | **Decreased#:** Dihydrouracil, Desaminotyrosine, Guanidinosuccinic acid, Methylguanidine.<br>**Increased#:** Valine, Dihydroxy-1H-indole glucuronide I, N-α-acetyl-l-arginine, lndoxyl, Norvaline, 5-Aminopentanoic acid, α-N-phenylacetyl-l-glutamine, P-cresol sulfate, Glucuronic acid Indoxyl sulfate, N-acetylasparagine. | Fatty acids β Oxidation, Mitochondrial dysfunction, Oxidative stress, Amino acid metabolism, Intestinal microbiota dysfunction |
| 22 | Gevi et al. (2016)[237] | Urine | HILIC-UHPLC | **Decreased#:** N-acetyl-5-methoxy tryptamine, Melatinin, Kynurenic acid.<br>**Increased#:** indolyl-3-acetic acid indolyl lactate, Inosine, Hypoxanthine, Xanthine, Xanthurenic acid, Quinolinic acid. | Vitamin B6, Reduced synthesis of melatonin, Intestinal ecological imbalance, Tryptophan and Purine metabolism pathways |
| 23 | Xiong et al. (2016)[238] | Urine | GC/MS | **Increased#:** 3-Hydroxyphenylacetic acid, 3-Hydroxyhippuric acid, 3-(3-Hydroxyphenyl)-3 hydroxypropionic acid. | Intestinal microbiota composition, Gastrointestinal symptoms, Urinary excretion |
| 24 | Lussu et al. (2017)[239] | Urine | 1H NMR Spectroscopy | **Decreased#:** Lactate, Valine, Betaine, Taurine, Glutamate, Creatinine.<br>**Increased#:** Glycine, D-threitol, Hippurate, Tryptophan. | Oxidative stress conditions, Gut microbiota modification |

| | | | | | |
|---|---|---|---|---|---|
| 25 | Bitar et al. (2018)[240] | Urine | LC-MS, NMR | **Decreased#:** N-methylglutamate, Creatine, Glucose-1-phosphate, Tyrosine, Urocanic acid, Cysteic acid, Citric acid, Guanine, Acetylcarnitine, Nacetylalanine, Serine, Threonine, Hydroxybenzoic, Hydroxyproline. **Increased#:** Riboflavin, Cholic acid, Phosphoserine, Glutamic acid, Trigonelline, 5-Aminioimidazole-4-carboxamide. | Intestinal microbiota, Energy metabolism, Oxidative stress, Purine and creatine metabolism |
| 26 | Chen et al. (2019)[218] | Urine | GC-MS, XGBoost | **Decreased*:** 3-Oxoglutarate, 3-Oxoglutaric, Aconitic, Phosphoric acid, Carboxycitric acid, Phosphoric acid. **Increased*:** Phenylactic acid. | Amino acid metabolism, Gut microbiota, Energy metabolism, Bone salt metabolism. |
| 27 | Gevi et al. (2020)[241] | Urine | UHPLC-MS | **Decreased#:** GABA, MHPG, P5P, VMA, Noradrenaline, Adrenaline, Vanillylmandelic acid. **Increased#:** Dopamine, Vitamin C, 4-Cresol, HVA. | Intestinal microbiota composition, Neurotransmission, Biological composition, DNA hypomethylation |
| 28 | Liang et al. (2020)[242] | Urine | 1H NMR Spectroscopy | **Decreased#:** Trigonelline, Melatonin, Pantothenate, Serotonin, Taurine. **Increased#:** Tryptophan, Hippurate, Glycine, Creatine | Changes of cysteine metabolism, Oxidative stress, Thiometabolism, Melatonin production |
| 29 | Anna et al. (2022)[243] | Urine | LC-MS/MS | **Decreased#:** Phenylalanine, Quinolinate, Asparagine, Piridoxyne, Methyl-histidine, Xanthosine, Uridine,Ornithine, D-glucarate, Tyrosine. **Increased#:** Hypoxantine, Guanine, Cystine, Acetylysine, Thiamine-phosphate, Deoxyribose-phosphate, Phenylpyruvic, Phenylacetic acid. | Intestinal microbiota derived compounds, Reduction and oxidative, metabolism of gut bacteria, Vitamin B6 and B12 |
| 30 | Sharon et al. (2019)[184] | Intestinal | GC-MS | **Decreased*:** 5-AV, GABA, Taurine. **Increased*:** 3AIBA, Soy-derived isoflavones, Genistein, Daidzein | Metabolites of intestinal bacteria, E/I balance, Neuronal development |
| 31 | Lu et al. (2021)[183] | Intestinal | LC-MS/MS | **Decreased*:** 6-Hydroxymelatonin, Indole-3-lactic acid, 5-Methoxy-Indoleacetate. **Increased*:** Trp-Trp, Kynurenic acid, Indole-3-acetic acid | Changes in gut microbiota, Tryptophan and Serotonin Metabolism |

**Abbreviations:** DMA: dimethylamine, DEHA: dehydroepiandrosterone sulfate, DHF: dihydrofolic acid, GABA: γ- aminobutyric acid, HC: healthy children, HVA: homovanillic acid, LPA: lysophosphatidic acid; LC-MS/MS, liquid chromatography-mass spectrometry/mass spectrometry; TPP: thiamine pyrophosphate, TD: typical development, Trp-Trp: a dipeptide of tryptophan, SCFA：short-chain fatty acid, NAG: N-acetyl glycoprotein fragments, NMNA: N-methyl nicotinic acid, MHPG: 3-methoxy-4-hydroxyphenethyleneglycol, NMND: N-methyl-nicotinamide, PAG: phenylacetylglutamine, P5P: pyridoxal phosphate, VMA: vanillylmandelic acid, 2PY: N-methyl-2-pyridone-5-carboxamide, 5-AV: 5-aminovaleric acid, 3AIBA: 3-aminoisobutyric acid, 4EPS: 4-ethylphenyl sulfate, 5MTHF: 5-methyltetrahydrofolate, 5-HT: 5-hydroxytryptamine.
